# Molecular and structural insights into carvacrol and thymol alkylated derivatives targeting WSSV and AHPND-causing *Vibrio parahaemolyticus*

**DOI:** 10.1007/s00203-026-04982-8

**Published:** 2026-06-06

**Authors:** Joao Miguel Lopes de Melo Lima, Damião Sampaio de Sousa, Carminda Sandra Brito Salmito-Vanderley, Emmanuel Silva Marinho

**Affiliations:** 1 Faculty of Veterinary Medicine, State University of Ceará, Fortaleza, CE Brazil; 2 Postgraduate Program in Veterinary Sciences, State University of Ceará, Fortaleza, CE Brazil; 3 Postgraduate Program in Natural Sciences, State University of Ceará, Fortaleza, CE Brazil

**Keywords:** Aquaculture, Shrimp Diseases, Molecular Modelling, Sustainable Drug

## Abstract

The intensification of shrimp farming has increased the incidence of infectious diseases associated with environmental instability, particularly White Spot Syndrome Virus (WSSV) and acute hepatopancreatic necrosis disease (AHPND), caused by *Vibrio parahaemolyticus*, resulting in high mortality and significant economic impacts. Given the limited efficacy of available therapeutic approaches, the search for bioactive compounds of natural origin emerges as an innovative and sustainable strategy. In this study, carvacrol and thymol derivatives were investigated through computational screening against the target proteins PirB (*V. parahaemolyticus*) and thymidylate synthase (WSSV). The results revealed energetically favorable binding profiles and high conformational stability of the complexes with PirB, as established by cavity analyses, suggesting strong antibacterial inhibitory potential. Although the affinity parameters for thymidylate synthase indicate lower antiviral priority, the consistent overlap with the co-crystallized ligand supports the plausibility of relevant biological activity. Notably, the carvacrol and thymol derivatives CVC and TMC compounds exhibited superior performance, demonstrating greater structural stability and interaction robustness. Taken together, these findings highlight carvacrol and thymol derivatives particularly CVC and TMC as promising candidates, as they combine moderate binding affinity with consistent structural stability across the evaluated targets. These results provide a computational framework for the rational development of new therapeutic strategies in shrimp farming, contributing to more effective and sustainable approaches, while warranting further experimental validation.

**Graphical abstract:**

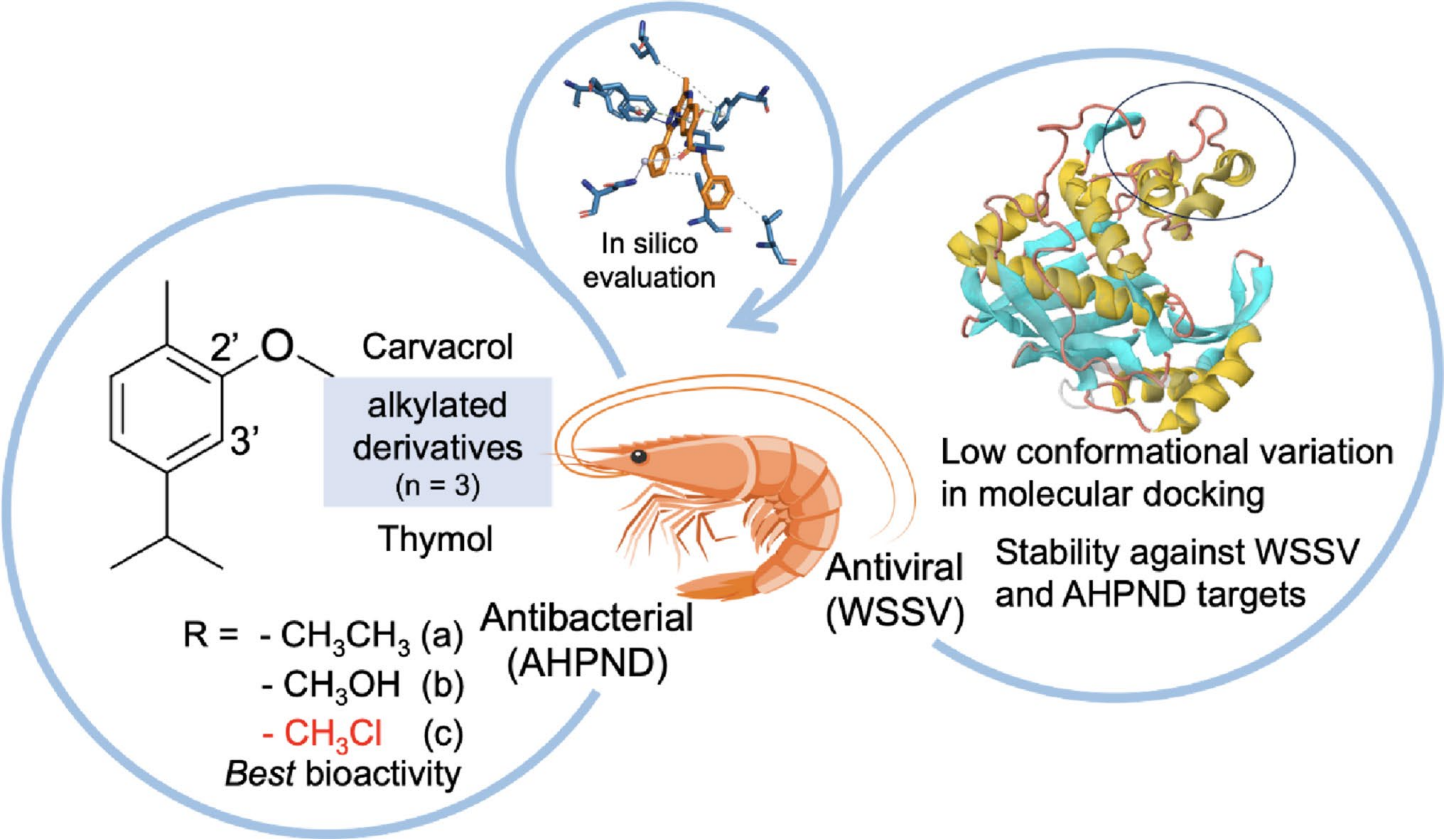

**Supplementary Information:**

The online version contains supplementary material available at 10.1007/s00203-026-04982-8.

## Introduction

The aquatic environment is shaped by multiple intrinsic factors, including pH, salinity, temperature, oxygen availability, and mineral composition, which collectively make it highly dynamic and difficult to control in aquaculture systems (Padilla et al. 2025). Even minor fluctuations can trigger stress in shrimp, affecting host susceptibility and overall health status, thereby increasing vulnerability to opportunistic pathogens such as White Spot Syndrome Virus (WSSV) and Acute Hepatopancreatic Necrosis Disease (AHPND), commonly associated with Vibrio parahaemolyticus (AHPND) (Gao et al. 2024).

WSSV, a member of the family Nimaviridae, exhibits a complex structural organization and a large double-stranded DNA genome (Rajendran et al. 2025). Its high virulence, systemic infections affecting multiple tissues, leading to rapid disease progression and mortality, environmental persistence, and multiple transmission routes pose significant challenges for disease management, while also causing cease of eating, lethargy, cuticle relaxation, and reddish discoloration, even before showing the white spots that name the syndrome (Chen et al. 2023a, b; De Sousa et al. 2025a, b). The wTS protein plays an essential role in the nucleotide synthesis pathway, catalyzing the conversion of dUMP to dTMP, a crucial step in DNA replication (Liu et al. 2025). In turn, the PirB (vp) protein is associated with bacterial virulence processes, interacting with host cellular components (Hao et al. 2019).

Outbreaks cause high mortality rates and substantial economic losses, as penaeid shrimp constitute a cornerstone of global aquaculture, with cumulative damages estimated in the billions of dollars (Lightner et al. 2012). Despite advances in biosafety, health management, and molecular diagnostics, no effective antiviral therapies are currently available for post-infection treatment. In this context, thymidylate synthase (wTS) was selected as a molecular target due to its central role in dTTP biosynthesis, a key precursor for DNA synthesis. As an essential enzyme for DNA replication, wTS is critical for viral proliferation. This underscores the urgent need for the development of novel preventive and therapeutic strategies (Raja et al. 2025).

Importantly, AHPND pathogenesis is driven by the expression of PirA/B toxins, encoded on plasmids, which function as a binary toxin system responsible for severe damage to hepatopancreatic tissues (Wang et al. 2026). Consequently, the virulence of *Vibrio parahaemolyticus* is tightly regulated by molecular mechanisms that modulate toxin expression and host interaction. Among these, the sensing of ferric iron (Fe³⁺) derived from the host acts as a key regulatory signal, directly triggering the activation of virulence-associated genes (Zhang et al. 2025a, b).

Also associated with mass mortality in shrimp farming, AHPND is primarily caused by the bacterium *Vibrio parahaemolyticus*, which is found in various countries in Asia and the Americas and has mortality rates exceeding 65% (Tang and Bondad-Reantaso 2019).

The disease presents nonspecific initial symptoms, similar to WSSV, such as lethargy and loss of appetite, but progresses to more characteristic manifestations, such as an empty stomach and midgut, culminating in a pale to whitish and atrophied hepatopancreas in the terminal stage’s signs associated with the disease’s high lethality (Fadel et al. 2025).

**Fig. 1:**
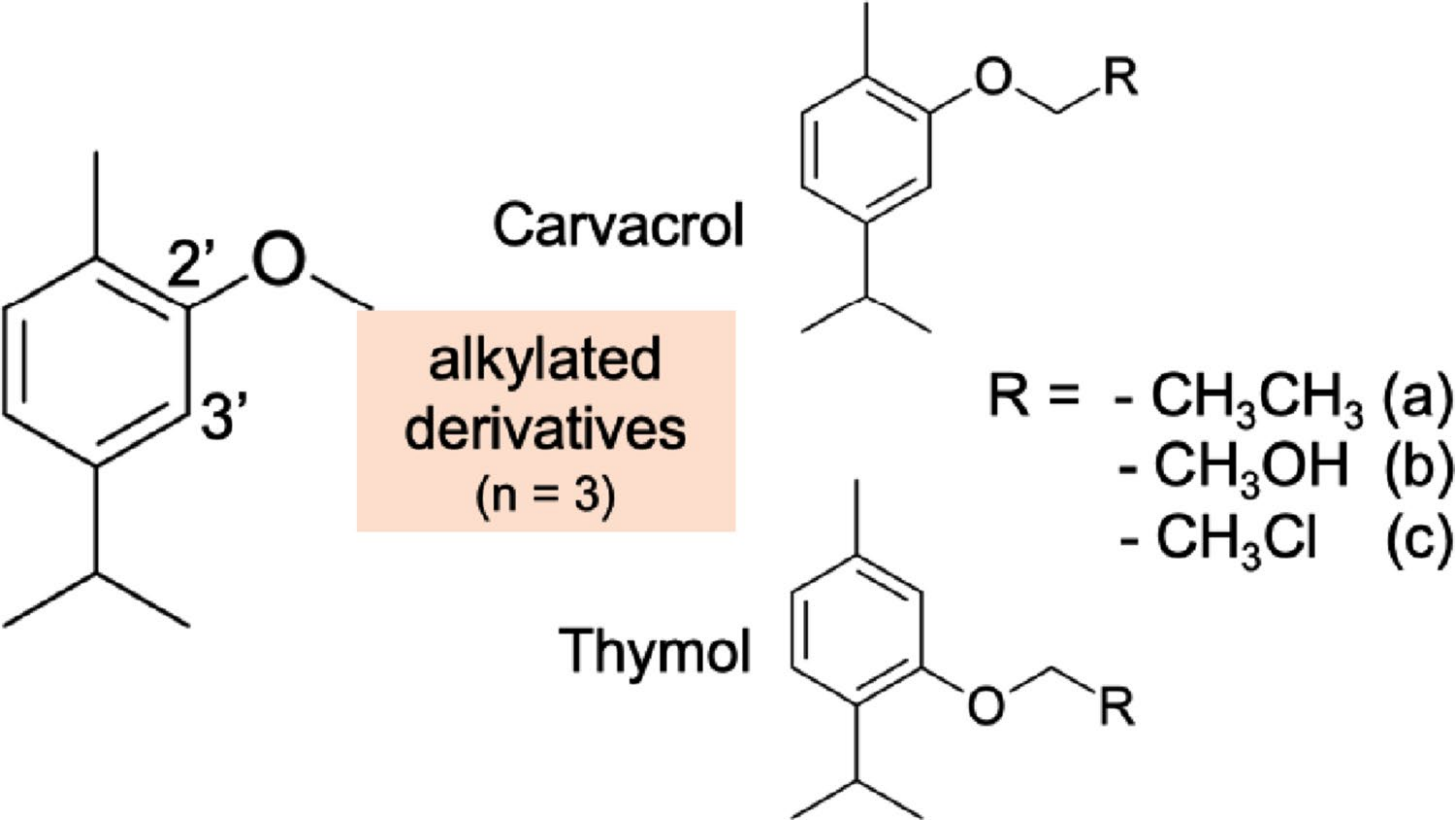
Representation of the two-dimensional structure of Carvacrol and Thymol molecules, and their respective derivatives. Adapted from (Natal et al. 2021).

Phenolic monoterpenes, particularly thymol and carvacrol, have emerged as versatile bioactive molecules (Rathod et al. 2021). Predominantly found in thyme and oregano, these structurally similar compounds exhibit comparable biological activities, including antiviral and broad-spectrum antibacterial effects against food-borne and aquaculture-related pathogens (Kachur and Suntres 2020; Miladi et al. 2016; Pathirana et al. 2021).

Structural derivatives of thymol and carvacrol have been synthesized to enhance stability, target affinity, and pharmacokinetic profiles, Fig. 1 (Natal et al. 2021). Computational analyses, including structure-based virtual screening and molecular dynamics simulations, suggest that these derivatives form stable interactions with target proteins, while nanoencapsulation in liposomes further improves stability and controlled release (Natal et al. 2021).

Building on these findings, the present study aims to evaluate the inhibitory potential of thymol and carvacrol-based derivatives against proteins associated with WSSV and AHPND through computational analyses. The goal is to predict interactions with relevant molecular targets and support the development of novel antiviral and antibacterial strategies based on structurally optimized natural products.

## Computational methodology

###  Biological activity profile of carvacrol/thymol derivatives

The biological activity profiles of carvacrol and thymol derivatives were predicted using the PASS Online platform (Prediction of Activity Spectra for Substances), available on the Way2Drug server (https://way2drug.com/PassOnline/). The analysis was performed based on the chemical structures of the compounds, entered in the appropriate format, allowing for the simultaneous prediction of multiple pharmacological activities or mechanisms of action (Filimonov et al. 2014).

The method employed by PASS is based on structural descriptors of the Multilevel Neighborhoods of Atoms (MNA) type and *Bayesian* algorithms to establish structure–activity relationships (SAR), estimating, for each activity, the probabilities of a compound being active (*Pa*) or inactive (*Pi*) (Lagunin et al. 2000).

1 $$ \Pr _{j} = \left( {1 + ~\left( {s_{j} - s_{{0j}} } \right){ / }\left( {1 - s_{j} \left. {s_{{0j}} } \right)} \right.} \right){ / }2 $$

$\:{s}_{j}$ composite score for activity *j*, based on the MNA descriptors. $\:{s}_{0j}$ training reference score (the threshold between active and inactive) and $\:{P}_{rj}$ final probability of activity, normalized between 0 and 1, and considered significant when Pa > Pi, Eq. 1.

In addition, the predicted activities were analyzed based on Pa and Pi values, with those having Pa > Pi considered biologically relevant, as higher Pa values indicate a greater probability of experimental confirmation (Filimonov et al. 1999). The platform exhibits an average prediction accuracy of approximately 95%, validated by leave-one-out cross-validation, which enables its applicability in virtual screening and the discovery of bioactive compounds (Filimonov et al. 2009).

### Molecular docking procedures

#### Structural optimization and molecular modeling of alkylated carvacrol/thymol derivatives

The major fragments and derivatives of carvacrol (CVA-C) and thymol (TMA-C), Fig. 1, were designed using MarvinSketch software (Laxmi 2022), making it possible to identify the physiological species and, consequently, the most stable conformer for each molecule. The optimization of these ligands was performed using the tools available in the Avogadro program (Hanwell et al. 2012). In this context, specific settings are used to ensure the best possible optimization, in which simulations are performed under the MMFF (Merck Molecular Force Field 94) force field, using the *Steepest Descent* algorithm and performing 50 numerical steps, with a defined convergence parameter (Jász et al. 2019).

#### Preparation and optimization of the structures of WSSV and AHPND (*V. parahaemolyticus*)

The three-dimensional structures were retrieved from the RCSB Protein Data Bank (https://www.rcsb.org/), entitled “Structure of WSSV thymidylate synthase in complex with dUMP” (Panchal et al. 2021) and “Crystal structure of PirB” (Lee et al. 2015) delimited with the information in Table 1, respectively, (Goodsell et al. 2020).

**Table 1:** Preparation of protein wTS and PirB(vp) methodology

Enzyme	PDB ID	Organism	Classification	Resolution	Citation
wTS	7XYJ	WSSV (isolate Tongan)	Transferase	2.27	(Panchal et al. 2021)
PirB(vp)	3 × 0U	*V. parahaemolyticus* M0605	Toxin	1.70	(Lee et al. 2015)

Overall, both structures were obtained by X-ray diffraction and are expressed in heterologous systems, with experimental parameters suitable for molecular modeling studies. Additionally, unwanted residues, such as solvent molecules and co-crystallized ligands, were removed using UCSF Chimera software (Butt et al. 2020), following the methodological guidelines described by (Da Silva et al. 2026b; Gomes et al. 2025).

### Molecular docking simulations

Molecular docking calculations were performed using AutodockVina software (Morris et al. 2009), to predict the modes of interaction and binding affinity between ligands and the active site of the target protein. For the conformational search, the *Iterated Local Search* was employed (Morris et al. 1998), which combines genetic operators with local optimization, thereby increasing the efficiency of identifying energetically favorable conformations.

**Table 2:** Criteria and procedures of molecular docking

	Gridbox (coordinates)^a^			Charges		Criteria (molecular docking)	
Enzyme	Center	Size	Inhibitor	*Gasteiger*	*Kollman*	EA^b^	RMSD^c^
wTS (136.66 kDa)	*x* (14.927) *y* (47.368) *z* (46.778)	(114) *x* (118) *y* (106) *z*	uMP^d^	+++	+++	na	≤ 2Å
PirB(vp) (102.52 kDa)	*x* (28.107) *y* (−3.753) *z* (26.633)	(114) *x* (126) *y* (1062) *z*	GEN^e^				

(a) Space covering the biomolecule. (b) Affinity energy (kcal/mol). (c) Root Mean Square Deviation (Å). (d) 2’-deoxyuridine 5’-monophosphate and (e) gentamicin (comparative control). na. not applicable

Accordingly, an *Exhaustiveness* value of 64 was adopted, ensuring a broader exploration of the conformational space and greater robustness in the results obtained (Roberto et al. 2025). The simulation parameters, including the number of independent runs, population size, maximum number of energy evaluations, and convergence criteria, were defined as described in Table 2.

Thus, the best poses were selected based solely on the Root Mean Square Deviation (RMSD, ≤ 2 Å) and the consistency of the observed interactions between the ligand and the receptor (de Oliveira et al. 2024).

The molecular docking protocol was validated through redocking procedures of the co-crystallized ligands, with the aim of evaluating the accuracy and reproducibility of the method in predicting binding poses (De Sousa et al. 2026). To this end, the uMP ligand was removed from its crystallographic structure and subsequently redocked at the active site of the wTS enzyme, while the GEN compound was redocked at the PirB (vp) protein, using methodological adaptations specific to this system.

Gentamicin (GEN) was included as a reference ligand due to its well-established antibacterial activity (Athauda et al. 2024). It was used as a control to support the docking protocol and to provide a comparative framework for evaluating ligand–protein interactions. The compound was subjected to the same preparation and docking procedures as the evaluated derivatives, allowing for a consistent comparison of binding affinity and interaction profiles.

This validation step involves verifying the protocol’s ability to reproduce the experimental conformations of ligands (crystallographic poses) at the binding site, serving as an indicator of the accuracy of the method employed. Thus, the results obtained corroborate the robustness of the computational approach adopted for predicting binding affinities and analyzing the selectivity profiles of organic compounds and their derivatives, lending greater reliability to subsequent docking simulations.

### Inhibitor potential vs. affinity energy

The inhibition potential of carvacrol and thymol derivatives was evaluated based on the binding energies (adapted, E_A_) of each ligand–protein complex, obtained through independent docking simulations. Based on these energies, the inhibition constants (K_i_) were calculated using the following equation, which relates the binding free energy to the interaction strength between the ligand and the protein’s active site (Silva et al. 2022).

2 $$\:Ki={e}^{\left(\frac{\varDelta\:G}{RT}\right)}$$

Where, Ki represents the inhibition constant, T represents the absolute temperature (298 K), R represents the universal gas constant (1.987 kcal·mol^− 1^·K^− 1^), and ΔG (adapted in E_A_) represents the free binding energy (kcal/mol), Eq. 2, (Kadela-Tomanek et al. 2021).

### Pharmacophore modeling and analysis

Predictions of the pharmacophore characteristics of the selected molecules were performed using the online platform Pharmit (https://pharmit.csb.pitt.edu/), ensuring the preservation of the stereochemistry, spatial orientation, and potentially bioactive conformation of each compound (Giordano et al. 2022).

In this process, the structures used in this study were subjected to the identification of pharmacophore descriptors, including functional donor and acceptor sites for hydrogen bonding, hydrophobic regions, and π-aromatic centers, allowing for the determination of the most relevant structural fragments (Sunseri and Koes 2016).

Based on this analysis, it became possible to assess the pharmacophore similarity between carvacrol and thymol derivatives and the ligands used in the validation redocking, providing insights into the structural elements critical for interaction with the active sites of the target proteins and informing strategies for the design of new bioactive compounds (Gonçalves et al. 2026).

### NMA-based molecular dynamics simulations

Molecular dynamics simulations based on Normal Mode Analysis (NMA) were performed for protein–ligand complexes involving the 7XYJ (wTS) and 3 × 0U (PirB, vp) proteins with carvacrol and thymol derivatives, as well as with the reference ligands uMP, respectively. The analyses were conducted on the iMODS server (https://imods.iqf.csic.es/) (López-Blanco et al. 2014).

The main parameters analyzed include: (i) Deformability of the binding regions, indicating the protein’s ability to adapt to the presence of the ligand (Cα RMSD); (ii) Calculated B-factors, providing estimates of atomic mobility throughout the structures; (iii) Global protein motion, represented by dominant normal modes, which highlight the main degrees of structural freedom; and (iv) Ligand-induced conformational stability, allowing the correlation of ligand presence with changes in protein rigidity or flexibility, (ΔCα RMSD) (López-Vallejo et al. 2011).

In this context, this approach complemented the results of molecular docking, providing a dynamic view of ligand–protein interactions and enabling the identification of which compounds confer greater stability or flexibility to the functional regions of the target proteins, thereby aiding in the prioritization of candidates with greater biological potential (Da Silva et al. 2026a).

### Ecotoxicological aspects of carvacrol/thymol derivatives

#### Quantitative structure-activity relationship (QSAR) based toxicity prediction

Quantitative Structure-Activity Relationship (QSAR) models are indispensable for the computational assessment of the ecotoxicology of the compounds used, making it possible to predict the toxicological, environmental, and ecotoxicological properties of chemical compounds, such as persistence and toxicity (Ghorpade-Aher et al. 2025).

Using the JANUS software, the molecules were evaluated and organized in Table 3 for analysis. The model adopted by JANUS employs a continuous metric ranging from 0 to 1, where values close to 0 indicate low risk, while values close to 1 indicate a higher potential for danger (Roncaglioni et al. 2022).

Additionally, conventional parameters (see Table 3) were used as a methodological reference for the analysis, with the aim of ensuring the reproducibility of the results in other software and guaranteeing alignment with the guidelines established by the Environmental Protection Agency, EPA (De Sousa et al. 2026).

**Table 3:** Toxicity classification based on the concentration required to kill 50% of the population under acute and chronic exposure, adapted from De Souza et al. (2026)

Toxicity Classification	Acute toxicity (mg/L)	ChV (mg/L)
High	< 1.0	< 0.1
Moderate	1.0–100	0.1–10
Low	> 100	> 10

To obtain the values used, the software employs two equations. Equation 3 is applied to estimate the acute toxicity of compounds not present in the database, expressed as a mathematical relationship between the logarithm of the LC_50_ concentration (mmol/L) and the logarithm of the partition coefficient (logKow), where *m* and *b* correspond to the coefficients of the regression model (De Sousa et al. 2026).

3 $$\:y=mx\:+\:b$$

4 $$ ChV = \log \left( {LOEC~ \cdot ~NOEC} \right){ / }~2 $$

Equation 4, in turn, calculates the geometric mean between the lowest concentration at which an effect is observed (LOEC) and the highest concentration at which no effect is observed (NOEC), resulting in an estimate of chronic toxicity (ChV) (De Sousa et al. 2025a, b).

#### Persistence assessment

The JANUS platform also allows for the assessment of the environmental persistence of compounds in the various compartments of the aquatic environment (water, sediment, and soil), based on QSPR and QSAR mathematical models (Burden et al. 2016).

**Table 4:** Ranking of persistence in days according to each compartment of the aquatic environment

Persistence Classification	Water	Sediment	Soil
nP	< 40	< 120	< 120
P	40–60	120–180	120–180
vP	> 60	> 180	> 180

The persistence classification is categorized as non-persistent (nP), persistent (P), and very persistent (vP), with thresholds defined according to the environmental compartment being assessed (Voigt and Jaeger 2024). The persistence categories, as well as the respective compartments considered for each classification, are presented in Table 4.

It is important to highlight the predictive nature of the screening performed in this study, whose methodology is based on the use of semi-empirical software for analysis. This approach does not replace the need for in vitro and in vivo assays to validate hypotheses, but serves as a classification and guidance tool, contributing to the prioritization of compounds and the optimization of the drug discovery process.

## Results and discussion

### Bioactivity in carvacrol/thymol derivatives

The analysis conducted using PASS Online was used exclusively as a preliminary screening tool, with the aim of identifying potential biological activity profiles for carvacrol, thymol, and their derivatives. In general, the observed low-to-moderate probability of activity (Pa) values underscore the predictive nature of this study. Consequently, these results are interpreted as preliminary indicators of biological potential, establishing a computational baseline to guide future experimental validation of carvacrol and thymol derivatives.

Thus, it was observed that most of the predicted activities had values below 0.7, a threshold generally associated with a higher probability of biological activity. In this regard, the data obtained do not allow us to confirm the actual presence of activity, but merely suggest trends based on structural similarity to previously described compounds, Table 5.

With regard to antibacterial activity, the Pa values were consistently low for all compounds evaluated, including carvacrol (0.319) and thymol (0.336), which are widely described in the literature as effective antimicrobial agents, Table 5 (Peter et al. 2024). This apparent inconsistency can be explained by the inherent limitations of the predictive model used. PASS relies on structural databases and previously reported activity patterns, which may not adequately capture nonspecific mechanisms of action, such as the disruption of cell membranes, a central feature of the activity of phenolic compounds (Filimonov et al. 2014).

**Table 5:** Targets and biological activity (p.a.: active and *pi*: inactive) of carvacrol/thymol derivatives

Compd	Antimycobcterial	Lactase inhibitor	Anesthetic	Antiviral	Antibacterial
	Pa/Pi	Pa/Pi	Pa/Pi	Pa/Pi	Pa/Pi
Carvacrol	0.449/0.028	0.459/0.038	0.433/0.015	0.356/0.057	0.319/0.053
CVA	0.362/0.050	0.347/0.094	0.645/0.005	0.352/0.059	na
CVB	0.365/0.050	0.513/0.025	0.575/0.007	0.347/0.0012	0.192/0.125
CVC	0.284/0.091	0.252/0.208	0.482/0.012	0.455/0.045	na
Thymol	0.473/0.023	0.459/0.038	0.502/0.010	0.454/0.031	0.336/0.047
TMA	0.389/0.042	0.347/0.094	0.710/0.004	0.531/0.016	0.171/0.145
TMB	0.392/0.041	0.513/0.025	0.631/0.005	0.533/0.015	0.208/0.110
TMC	0.306/0.077	0.252/0.208	0.547/0.008	0.472/0.036	na

Thus, the low probability predicted for antibacterial activity does not necessarily reflect an absence of biological effect, but rather a limitation of the model in recognizing mechanisms that do not depend on specific molecular interactions with well-defined targets (Poroikov et al. 2000). This aspect is particularly relevant for compounds such as carvacrol and thymol, whose action is often associated with physicochemical properties, such as lipophilicity and the ability to interact with lipid bilayers (Gandova et al. 2023).

With regard to antiviral activity, moderate Pa values were observed, particularly for some thymol derivatives (~0.53), see Table 5. Although these results may indicate a possible trend of activity, they should not be interpreted as evidence of antiviral efficacy. Rather, they suggest that certain structural modifications may bring these compounds closer to chemical classes previously associated with antiviral activities in the model database (Poroikov et al. 2019).

Another important finding was the presence of additional predicted activities, such as anesthetic effects and lactase inhibition, with moderate values observed in some derivatives (Bianchini et al. 2017; Zhang et al. 2023a, b). Although these activities are not directly related to the study’s objective, they demonstrate the multifunctional nature of phenolic compounds and highlight the breadth of the predictions generated by PASS, which should be interpreted with caution.

In general, the variations observed between parent compounds and their derivatives indicate that structural modifications directly influence the predicted activity profile (Druzhilovskiy et al. 2017). These changes can affect parameters such as polarity, electron distribution, and molecular volume, thereby influencing how the algorithm classifies the compounds. However, such changes primarily reflect adjustments in computational similarity, rather than actual improvements in biological activity (Dmitriev et al. 2019).

### Correlation between inhibition potential and affinity energy

#### Inhibition potential and affinity energy via *V. parahaemolyticus*

The results obtained for the PirB–derivative complexes allow a comparative analysis of the relationship between binding energy (E_A_), estimated inhibition constant (pK_i_), and structural deviation (RMSD). The binding energy (E_A_) ranged from −7.3 kcal/mol (GEN, reference) to −5.7 kcal/mol (TMA), reflecting variations in the predicted interaction profiles of the compounds within the protein binding site.

In general, a strong direct correlation was observed between E_A_ and pK_i_ (*r* = 0.9905), consistent with the expected mathematical relationship between binding energy and the estimated inhibition constant (K_i_). Compounds with more negative E_A_ values, such as GEN (−7.3 kcal/mol) and CVC (−6.3 kcal/mol), exhibited higher estimated pKi values (5.46 and 4.64, respectively), indicating relatively stronger predicted interactions. In contrast, compounds with less negative E_A_, such as TMA (−5.7 kcal/mol) and thymol (−5.8 kcal/mol), showed lower pK_i_ values (4.30–4.35), reflecting comparatively weaker predicted interactions. This trend supports the internal consistency of the computational estimates across the evaluated compounds (see Table 6), (Silva et al. 2022).

However, the analysis of RMSD provides additional structural context. RMSD values below 2 Å, observed for all complexes, indicate that the ligand poses remain close to their initial docked conformations. Nevertheless, compounds such as CVA and CVB exhibit relatively higher RMSD values (1.909 and 1.853 Å), despite having E_A_ and pK_i_ values comparable to other derivatives, see Table 6. This behavior may reflect differences in ligand flexibility or local conformational adjustments within the binding region, describing that interaction profiles are influenced not only by energetic estimates but also by structural accommodation (Du et al. 2016).

**Table 6:** Thermodynamic, energetic, and conformational descriptors of carvacrol/thymol derivatives via PirB(vp)

Complex/PirB	E_A_ (kcal/mol) ^b^	K_i_ (M) ^c^	pK_i_^d^	RMSD (Å) ^e^
GEN^a^	−7.3	3.47 × 10^− 6^	5.46	1.136
Carvacrol	−6.2	2.57 × 10^− 5^	4.59	1.388
CVA	−6.1	3.03 × 10^− 5^	4.52	1.909
CVB	−6.0	3.57 × 10^− 5^	4.45	1.853
CVC	−6.3	2.31 × 10^− 5^	4.64	1.654
Thymol	−5.8	4.44 × 10^− 5^	4.35	1.077
TMA	−5.7	5.02 × 10^− 5^	4.30	1.532
TMB	−5.9	3.93 × 10^− 5^	4.41	1.226
TMC	−6.0	3.57 × 10^− 5^	4.45	1.419

a. Comparative control. b. Affinity energy. c. inhibition constant; d. inhibition potential (logarithm of the inhibition constant, Ki) and e. Root Mean Square Deviation (RMSD)

Although the correlation between E_A_ and pK_i_ is strong, small variations can be observed when structural parameters are considered. For example, CVC exhibits a more negative E_A_ (−6.3 kcal/mol) and a slightly higher pK_i_ (4.64) compared to CVB (−6.0 kcal/mol, pK_i_ 4.45), while also presenting a lower RMSD (1.654 Å vs. 1.853 Å). This observation suggests that more consistent binding poses may be associated with slightly more favorable predicted interaction profiles, although such differences should be interpreted within the limitations of docking-based estimations.

Overall, compounds such as GEN and CVC display comparatively more favorable interaction profiles with PirB, combining lower (more negative) E_A_ values with consistent docking poses. In contrast, thymol and its derivatives (TMA, TMB, TMC) exhibit less negative EA and lower estimated pK_i_ values, but still maintain RMSD values below 1.6 Å, indicating that their predicted binding poses remain structurally consistent within the docking framework, see Table 6.

#### pK_*i*_ vs. E_A_ via wTS

Analysis of the complexes with wTS (thymidylate synthase) revealed variations in binding energy (E_A_), estimated inhibition constants (pK_i_), and structural deviation (RMSD) among the evaluated ligands. The uMP (co-crystallized ligand) exhibited the most favorable predicted interaction profile (E_A_ = − 7.6 kcal/mol; pK_i_ = 5.58), serving as a reference within the studied set (see Table 7).

**Table 7:** Thermodynamic, energetic, and conformational descriptors of carvacrol/thymol derivatives via wTS

Complex/wTS	E_A_ (kcal/mol)^b^	K_i_ (M)^c^	pK_i_^d^	RMSD^e^ (Å)
uMP^a^	−7.6	2.64 × 10^− 6^	5.58	1.438
Carvacrol	−5.8	4.44 × 10^− 5^	4.35	1.723
CVA	−5.8	4.44 × 10^− 5^	4.35	1.405
CVB	−5.5	6.15 × 10^− 5^	4.21	1.636
CVC	−5.9	3.93 × 10^− 5^	4.41	1.412
Thymol	−5.2	8.33 × 10^− 5^	4.08	1.128
TMA	−5.6	5.73 × 10^− 5^	4.24	1.688
TMB	−5.6	5.73 × 10^− 5^	4.24	1.688
TMC	−5.4	7.03 × 10^− 5^	4.15	1.638

a. co-crystallized ligand. b. Affinity energy. c. inhibition constant; d. inhibition potential (logarithm of the inhibition constant, Ki) and e. Root Mean Square Deviation

Phenolic derivatives, including carvacrol, thymol, and their analogues, showed less negative E_A_ values (−5.2 to −5.9 kcal/mol), corresponding to lower estimated pK_i_ values (4.08–4.41). These results indicate comparatively weaker predicted interactions with wTS relative to uMP, while still establishing the ability of these compounds to occupy the binding region within the docking framework (see Table 7).

Most derivatives exhibited RMSD values below 1.7 Å, indicating that the docked poses remain close to the initial binding conformation. Notably, carvacrol presented a slightly higher RMSD (1.723 Å) compared to CVA (1.405 Å) and CVC (1.412 Å) (see Table 7), which may reflect differences in ligand flexibility or local conformational adjustments within the binding site. This observation highlights that structural consistency of docking poses does not necessarily correlate directly with energetic estimates, reinforcing the need to consider both parameters in a complementary manner (Klebe 2026).

Furthermore, although the differences in E_A_ among the derivatives are modest, small variations are reflected in the corresponding estimated pK_i_ values, as supported by the strong correlation observed (*r* = 0.9920). For instance, CVC (E_A_ = − 5.9 kcal/mol) presents a slightly higher pK_i_ (4.41) compared to the other derivatives (see Table 7), suggesting subtle differences in predicted interaction profiles. These observations emphasize that comparative evaluation of ligands should integrate both energetic and structural parameters, while acknowledging the approximate nature of docking-derived estimates (Du et al. 2016).

### Molecular docking results

#### Redocking and structural evaluation of protein–ligand interactions between uMP and GEN

**Fig. 2:**
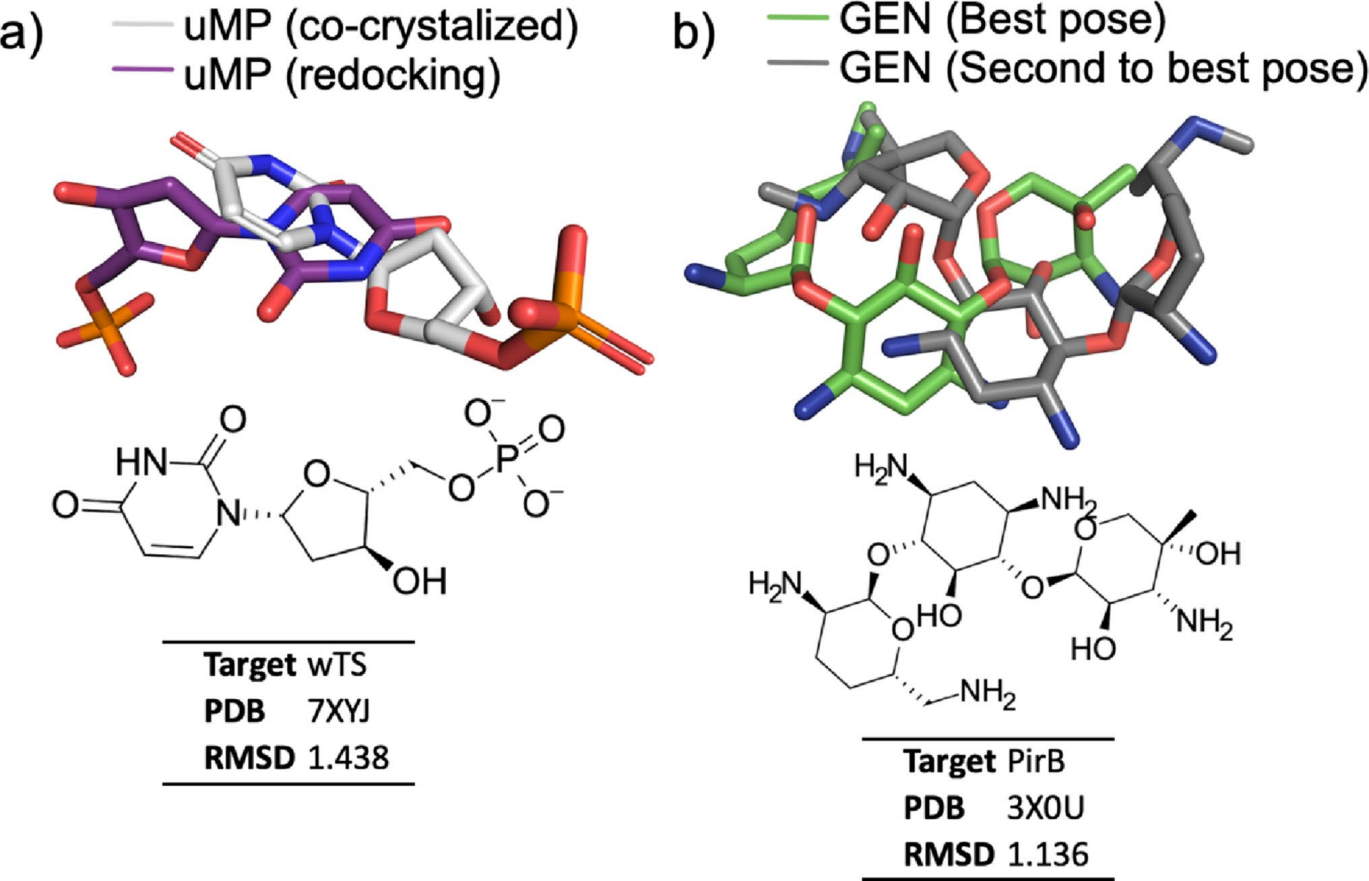
RMSD data from redocking ligands (purple and gray color) in relation to the co-crystallized to PDBs (white and green color): **a** uMP (wTS co-crystallized) and **b** GEN PirB (vp) inhibitor

The complex formed upon uMP–wTS redocking exhibited an affinity energy of −7.6 kcal/mol, a value consistent with stable interactions at the active site and capable of reproducing the orientation of the native ligand, Fig. S1a. The selectivity of the complex was supported by hydrogen bonds formed with H171, S191, and Y233, which play a decisive role in the orientation and positioning of the ligand within the catalytic cavity. The superposition between the co-crystallized uMP molecule and the one obtained from redocking confirms the conformational reproducibility of the system, Fig. 2a, validating the computational protocol employed. Additionally, the formation of multiple salt bridges with R24, R150, R151, and R190 indicates strong electrostatic interactions at the active site, contributing significantly to the stability and specificity of the complex, and acting as an additional level of stabilization beyond hydrogen bonds and hydrophobic interactions, Fig. S1a.

Gentamicin (GEN), used as a positive control due to its well-established antibacterial activity, exhibited a binding energy of −7.3 kcal/mol in the complex with PirB, indicating high affinity for the active site, Fig. S1b. The interactions are predominantly mediated by hydrogen bonds with Y201 and R236, in addition to an interaction with E289, which act as structural selectivity sites and direct the ligand into the protein pocket, Table 6. Concurrently, a significant set of hydrophobic interactions was observed with T209, A231, F244, L267, and T288, indicating that, following the simulations, the ligand accommodates itself in nonpolar regions of the active site, Table 6. Notably, the interactions with F244 and L267 indicate deep hydrophobic contact even in the absence of extensive aromatic groups in the gentamicin structure, highlighting high conformational adaptability, Table 8.

#### Protein–ligand interaction analysis of carvacrol/thymol derivatives with *V. parahaemolyticus*

When compared to carvacrol, significant changes in the binding mode are observed. Unlike GEN, carvacrol does not form hydrogen bonds and is stabilized predominantly by hydrophobic interactions with residues I130, V133, and L193, Table 8. This profile suggests that carvacrol does not interact with the polar site of the cavity in the same way as GEN, and may also indicate the occupation of distinct subregions within the binding site.

**Table 8:** Data on ligand-receptor (L-R) interactions in the redocking process of the comparative ligand GEN and molecular docking simulations of the carvacrol/thymol derivatives, via PirB(vp) receptor

Compd	Inter. type	Residues/Distances (Å)
GEN	Hydrophobic	T209B (3.72), A231B (3.99), F244A (3.67), F244B (3.92), L267A (3.99), T288B (3.69)
	H-bond	Y201A (2.52), R236B (3.02), E289B (3.56)
Carvacrol	Hydrophobic	Y129A (3.60), I130A (3.59), V133A (3.80), F185A (3.67), D189A (3.73), K192A (3.72), K192A (3.86), L193A (3.69)
CVC	Hydrophobic	F244A (3.65), F244A (3.58), F244A (3.69), M247A (3.64), L2267A (3.53), T 288B (3.55)
	π-Stacking	F244A (4.44)
Thymol	Hydrophobic	I130B (3.90), I130B (3.82), I138B (3.95), D189B (3.85), K189B (3.85), L193B (3.56)
	H-bond	K192B (2.63), N196B (2.09)
TMC	Hydrophobic	A213B (3.89), F244A (3.60), F244A (3.70), M247A (3.70), L267A (3.65), T288B (3.63)
	π-Stacking	F244A (4.30)

 Continuing the comparison with GEN, analysis of the carvacrol derivatives (CVA, CVB, and CVC) reveals a consistent change in the pattern of protein interaction. Unlike unsubstituted carvacrol, these derivatives combine multiple stabilization mechanisms, including orientation via hydrogen bonds, energetic anchoring through deep hydrophobic interactions, and additional contributions from π-stacking, see Table 8 and Table S1.

In this context, the CVA derivative forms an additional hydrogen bond with Y201, in addition to hydrophobic interactions with F244, M247, and L267, Table S1, suggesting a more efficient fit within the cavity compared to the original compound (Xiao et al. 2024). Complementarily, CVB stands out for forming hydrogen bonds with D216 and R236, associated with a π-stacking interaction with F244, Table S1. This interaction is particularly relevant, as it contributes to the stabilization of the ligand at the center of the cavity and reduces its conformational mobility (Langarizadeh et al. 2023).

Although CVA and CVB do not occupy the same binding site as GEN, the convergence in their interaction profiles suggests a potential synergistic effect, in which these compounds could act in a complementary manner to the drug, without directly competing for the binding site, Table S1.

**Fig. 3:**
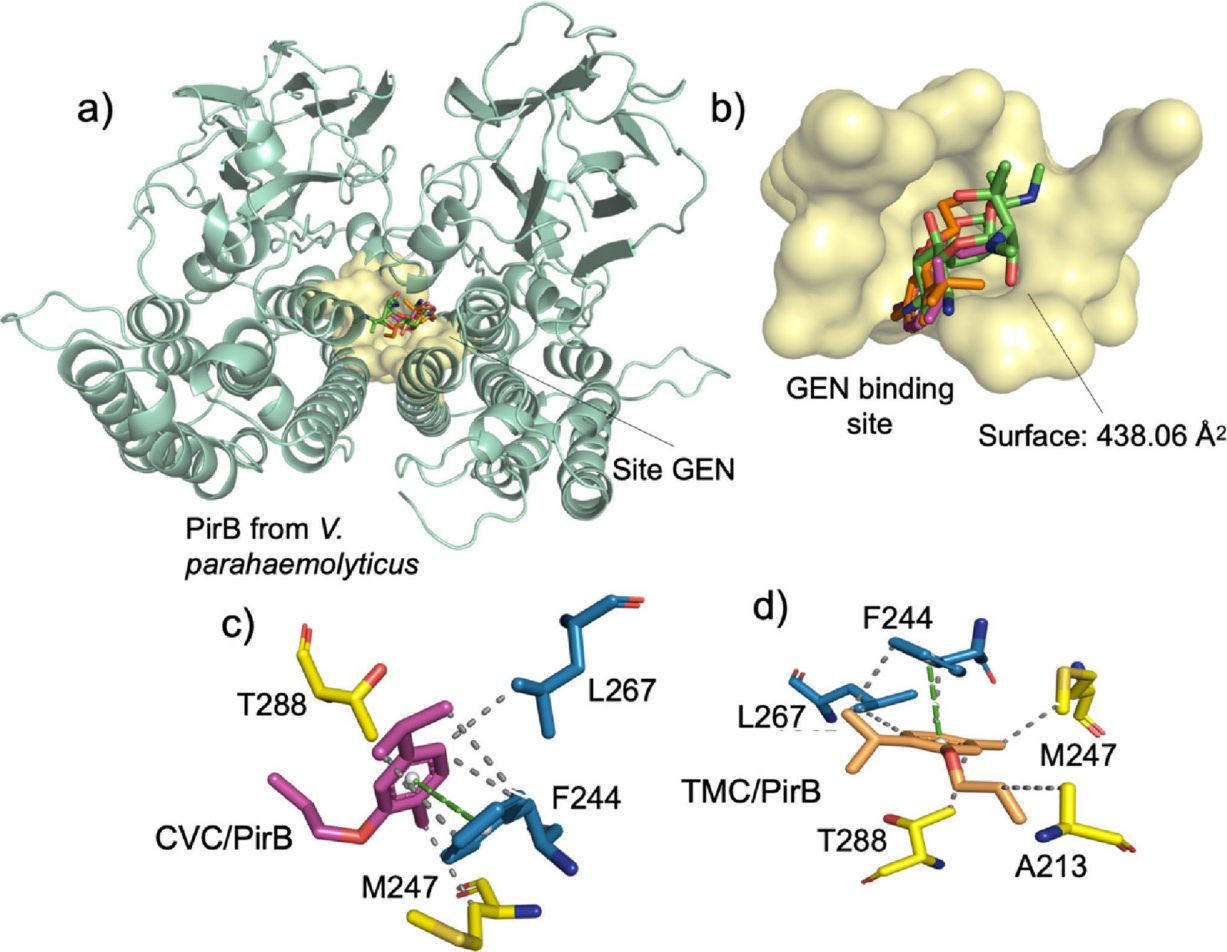
**a** Three-dimensional representation of the PirB/GEN (green), CVC (magenta), and TMC (orange) complex. **b** Interaction cavity of PirB, GEN, and derivatives. **c** L-P interactions mediated by CVC in molecular docking simulations. **d** L-P interactions mediated by TMC in molecular docking simulations (hydrophobic: gray and π-stacking: green)

In contrast to the other derivatives, CVC exhibited the lowest interaction energy, making it the most favorable compound within this group. These derivative forms hydrophobic interactions with F244, M247, and L267, as well as π-stacking interactions with F244, resulting in a more robust stabilization pattern, Table 6. The combination of deep hydrophobic contacts and aromatic interactions suggests that the ligand not only orients itself properly within the active site but also remains firmly accommodated, highlighting the decisive role of its structural architecture in stabilizing the complex (Zhang et al. 2023a, b).

This behavior can be attributed to the presence of an aromatic center capable of promoting π interactions, combined with hydrophobic regions enhanced by methyl groups at the ends of the molecule characteristics typical of phenolic monoterpenes as well as to the introduction of an aliphatic chain containing a chlorine heteroatom, which contributes to the formation of new hydrophobic regions, Fig. S2.

Thus, although gentamicin still exhibits higher overall affinity, CVC stands out as the carvacrol derivative with behavior most similar to the reference drug. This result is supported by the similarity in the interaction profile, see Fig. 3c, the spatial overlap observed in the cavity analysis, see Fig. 3b, and the structural convergence evidenced, see Fig. 3a, indicating potential functional equivalence in the context of biological activity.

Consistent with previous analyses, the simulations indicate that thymol and its derivatives exhibit an interaction pattern similar to that observed for carvacrol. Thymol, despite exhibiting lower affinity, largely reproduces the interaction profile of carvacrol, Table 8, differing, however, by the presence of hydrogen bonds, especially with residues Y192 and N196. These interactions contribute to the initial orientation of the ligand at the binding site, while the predominant stabilization remains associated with hydrophobic contacts with I130, I138, and L193, see Table 8.

This behavior suggests that thymol, similarly to carvacrol, may not occupy the same binding region as GEN. The absence of direct overlap between the predicted binding regions indicates a potential non-competitive interaction profile. However, this observation should be interpreted with caution, as it does not provide direct evidence of synergistic or potentiating effects (Sekar et al. 2024). Nevertheless, phenolic compounds have been reported to modulate antimicrobial activity in different contexts, and such interaction patterns may warrant further experimental investigation (Sreepian et al. 2022).

In contrast, the thymol derivatives (TMA, TMB, and TMC) generally exhibited interaction levels below the threshold considered ideal for a representative interaction with the 3 × 0U protein residues. The TMA derivative exhibited the lowest affinity, being stabilized predominantly by hydrophobic interactions with F244, M247, and L267, in addition to aromatic π-stacking, which suggests internal accommodation but with limited polar stabilization, Table S1.

In turn, TMB exhibited distinct behavior, establishing multiple hydrogen bonds with T201, S205, T209, and N217, indicating better structural orientation within the cavity. Nevertheless, this pattern did not translate into a significant energy gain. In contrast, TMC stood out as the only thymol derivative to achieve an energetically significant affinity level, exhibiting the lowest energy among its analogs. This compound combines multiple deep hydrophobic contacts with F244, M247, and L267, in addition to π-stacking interaction with F244, see Fig. 3d, a pattern consistent with its predominantly hydrophobic structural nature.

Furthermore, as a positional isomer of the CVC derivative, TMC shares similar pharmacophore characteristics, Fig. S3. The interactions observed in the TMC/PirB complex corroborate these properties, highlighting the significant role of the added aliphatic chain in modulating the complexes energy a finding consistent with the fact that TMC was the only derivative to achieve the ideal energy parameter (Chen et al. 2016, 2023a, b).

Therefore, the three derivatives exhibited partially overlapping positioning with that of gentamicin, particularly TMC, whose overlap is more pronounced. This result is consistent with its ability to interact with the same cavity occupied by GEN (see Fig. 3a), reinforcing the hypothesis that these compounds may exert antibacterial activity through protein inhibition.

Carvacrol and thymol are widely recognized for their antimicrobial activity, which is primarily associated with membrane disruption mechanisms (Ahmad et al. 2011). However, structural modification of these compounds may alter their physicochemical properties and interaction profiles, potentially enabling additional modes of action (Dong et al. 2024).

In this context, the docking results presented here should be interpreted as an exploratory assessment of possible protein–ligand interactions, rather than definitive evidence of target-specific inhibition (Akermi et al. 2024). Therefore, membrane-related effects and protein interactions should be considered as potentially complementary mechanisms, requiring further experimental investigation to determine their relative contributions (Marinelli et al. 2018).

Based on the available evidence, recent studies also report that the use of *Origanum* essential oils can reduce the virulence of *V. parahaemolyticus* associated with Acute Hepatopancreatic Necrosis Disease (AHPND) in *Penaeus vannamei*, indicating practical applicability in controlling outbreaks in aquaculture, with effects on both bacterial viability and pathogenic characteristics that determine the disease (Rodríguez et al. 2024).

Similarly, carvacrol has demonstrated effective inhibitory activity against aspects of *Vibrio cholerae* virulence, reducing mucin penetration, adhesion, and the expression of virulence-related genes, which resulted in a decrease in pathogenicity in experimental models. This positions carvacrol as a molecule of interest not only for killing or inhibiting growth, but also for reducing the expression of pathogenic factors in Vibrio species (Das et al. 2021).

In their study, Oramadike and Ogunbanwo (2017) report that ethanol extracts rich in thymol derived from *Thymus vulgaris* demonstrated antagonistic activity against *Vibrio parahaemolyticus* isolates from seafood, showing significant inhibition zones and a reduction in bacterial counts in direct experiments with the target species. These data highlight the role of thymol as an effective antimicrobial agent against relatively resistant strains of Vibrio.

Comparative studies of the chemical composition and biological activity of essential oils from various species of the Lamiaceae family show that oils in which thymol and carvacrol are the major components exhibit lower inhibitory concentrations against *V. parahaemolyticus* and other Vibrios, suggesting that these phenols contribute substantially to the observed antimicrobial activity (Dos Santos Filho et al. 2022).

Furthermore, comprehensive reviews of essential oil compounds as alternatives to antibiotics in aquaculture highlight that substances such as thymol and carvacrol have multiple mechanisms of action, including direct inhibition of bacterial growth, disruption of quorum sensing, reduction of biofilm formation, and interference with virulence factors, which is particularly relevant for the management of pathogens such as *Vibrio parahaemolyticus* in shrimp and fish farming environments (Srinivasan et al. 2025).

#### Interaction profiling of carvacrol/thymol derivatives with wTS

**Fig. 4:**
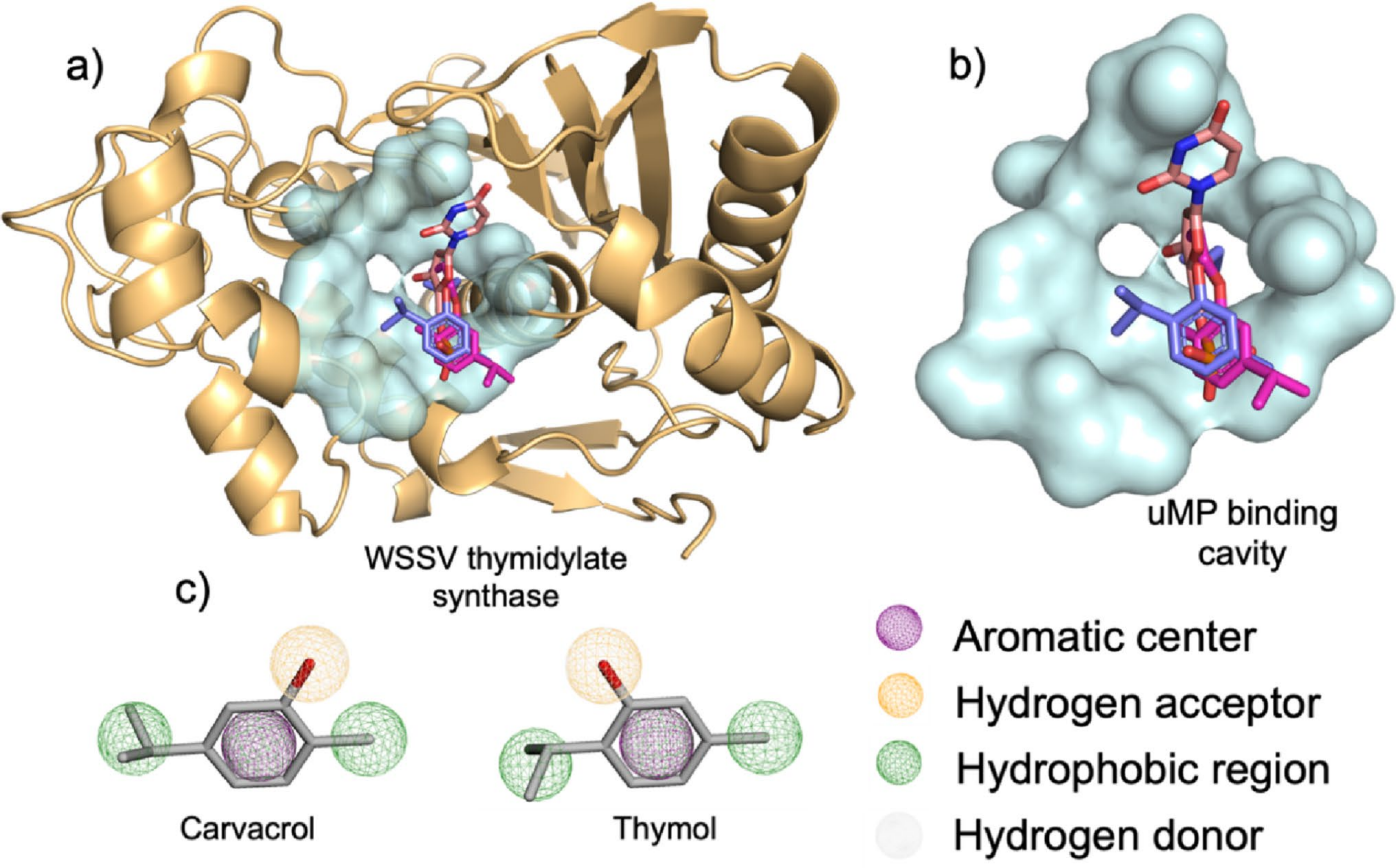
Molecular docking analysis. **a** Specific evaluation of carvacrol/thymol derivatives. **b** Interaction cavity of the wTS protein and **c** pharmacophore-based structural profile

 The docking results indicated that both the parent compounds (carvacrol and thymol) and their derivatives bind to the same active site, demonstrating a conserved pattern of selectivity, see Fig. 4a. However, the derivatives exhibited lower binding energies compared to the co-crystallized (uMP), resulting in reduced target selectivity, Table S2.

Interaction analysis showed that, while uMP formed multiple hydrogen bonds (H171, S191, Y233) and salt bridges (R24, R150, R151, R190), the derivatives interacted primarily via hydrophobic and π-stacking interactions, highlighting F200A, L196A, I83A, and W84A as recurring residues among the compounds, Table S2 (Panchal et al. 2021).

Among the derivatives, CVC and TMA were selected for NMA mode due to the interaction profile observed in docking: both formed hydrophobic and π-stacking interactions with critical residues such as F200A, L196A, and I83A, while partially maintaining hydrogen bonds (L196A and N201A), see Table S2 and Fig. 4c (Arvizu-Flores et al. 2009). Although they exhibit lower affinity compared to the parent compounds, the RMSD results indicate that the complexes maintain relatively consistent conformations throughout the simulation, suggesting that these molecules still possess modulatory potential by preserving strategic contacts at the active site.

Thus, the literature shows that phenolic plant compounds such as thymol and carvacrol possess immunomodulatory and antiviral properties that may influence their interaction with biological targets in aquatic organisms. The review conducted by (Dhama et al. 2018) concludes that plant secondary metabolites can interact with viral proteins and cellular modulators, providing theoretical support for the selectivity observed in the parent compounds.

Similarly, the study using microencapsulated thyme essential oil in *L. vannamei* demonstrates that thymol and carvacrol retain biological effects even after modifications (such as microencapsulation), showing that structural changes may reduce direct affinity but preserve essential interactions with the target (Tomazelli Júnior et al. 2018), which aligns with the pattern of hydrophobic and π-stacking interactions observed in the derivatives.

Furthermore, the review on the effects of thymol in fish and the study with olive leaf (*Olea europaea*) extract corroborate that phenolic compounds can preserve strategic contacts and modulate biological responses without relying exclusively on high-affinity interactions (Alagawany et al. 2021; Gholamhosseini et al. 2020), revealing the plausibility that thymol and carvacrol derivatives may still retain modulatory potential even with lower affinity energy.

The selected structure represents a catalytically relevant conformation of thymidylate synthase; however, as a homodimeric enzyme, its functional dynamics and inter-subunit interactions may influence ligand binding. Therefore, the present analysis is limited to a single structural state and should be interpreted as a static approximation.

Although the docking results indicate that the evaluated compounds are capable of interacting with wTS, the observed binding energies are comparatively modest and should be interpreted with caution.

These values do not support strong inhibitory effects, but rather suggest a limited interaction potential within the docking framework. In this context, the findings should be considered as preliminary and exploratory, highlighting possible interaction patterns rather than definitive antiviral activity. Therefore, further experimental studies are required to determine the actual biological relevance of these interactions.

## Pharmacophore-based analysis

Analysis of the interactions observed for the carvacrol group and its derivatives (carvacrol, CVA, CVB, and CVC) reveals a pattern consistent with that identified in docking simulations with the 3 × 0U (*V. parahaemolyticus*) protein. In general, hydrophobic contacts predominate with residues located both at the entrance and inside the cavity, notably I83, L196, and, recurrently, the aromatic residue F200, Table 6. The systematic interaction with this residue in all complexes points to its role as a key element of structural recognition at the active site (Ferreira et al. 2015), acting as a preferred region for the accommodation of the ligands’ phenolic ring.

Additionally, most derivatives exhibited π-stacking aromatic interactions with F200. This type of interaction occurs when the ligand’s aromatic ring aligns parallel to the phenylalanine ring, promoting electronic stabilization through π-density overlap (Salonen et al. 2011). The recurrence of this pattern highlights the insertion of the ligands into the hydrophobic core of the catalytic site, rather than merely a surface contact. Taken together, these findings indicate that the compounds not only bind to the protein but occupy regions critical for substrate recognition, which supports their potential as modulators of enzymatic activity (Chen et al. 2024).

The hydrophobic interactions observed contribute to the thermodynamic stabilization of the complex by excluding water molecules from the interior of the protein cavity (Sharma et al. 2021). This effect may compensate, to some extent, for the affinity energy values obtained for carvacrol, CVA, CVB, and CVC, which are close to the energy relevance threshold. In this context, although the formed L–P complexes exhibit structural stability, competition with higher-affinity ligands may limit the effective occupation of the active site, reducing the probability of establishing the most favorable interactions (Xu et al. 2025).

Complementarily, the evaluation of the interactions of the thymol group (thymol, TMA, TMB, and TMC) revealed a pattern largely consistent with that observed for the carvacrol derivatives. This convergence in the interaction profile indicates that both groups share similar structural and energetic characteristics, suggesting the possibility that they perform analogous functions in the context of the 7XYJ (wTS) protein, Table S2.

This behavior is likely associated with the high structural similarity among these compounds, as demonstrated by (Natal et al. 2021), who showed that positional isomerism has a limited influence on the pharmacophore characteristics of these molecules. In fact, both the compounds and their derivatives, as discussed earlier, have a predominantly hydrophobic structural core, resulting from the presence of methyl groups at the ends and the benzene ring characteristic of phenolic monoterpenes (see Fig. 3a) (Gabriel et al. 2024).

This hydrophobic character is directly reflected in the profile of interactions with the protein, since most contacts occur with nonpolar residues, such as leucine (L), isoleucine (I), phenylalanine (F), and threonine (T), involving both the central fragment and the substituents of the molecules. This interaction pattern is consistent with the high stability of carvacrol/thymol complexes with the 7XYJ protein, which is favored by the exclusion of water molecules from the cavity, resulting in energetically more favorable interactions (Vaidyanathan et al., 2023).

In addition, the aromatic center plays a central role in the stability of the complexes. Most of the molecules evaluated in this study formed π-stacking interactions, in which electronic stabilization occurs through π-density overlap, contributing to the maintenance of the complex and hindering its dissociation, in addition to restricting the access of new ligands to the active site, see Fig. 4c (Chen et al. 2016; Khatymov et al. 2025).

In this context, although the affinity energy values do not fully meet the parameters considered ideal, the combined administration of these compounds may compensate for such energetic limitations. The similarity in the interaction profile and the overlap observed in the cavity analysis indicate a potential synergistic effect, since the ligands interact with the same region occupied by the co-crystallized uMP (see Fig. 4b), suggesting a convergent action in the functional blocking of the active site.

Comparative analysis suggests that the improved interaction profiles observed for CVC and TMC may be associated with the presence of halogenated and aliphatic substituents, which enhance hydrophobic contacts within the binding cavity and favor more consistent accommodation of the ligand (Zhang et al. 2025a, b). These structural features appear to contribute to slightly more favorable energetic profiles and more consistent docking poses compared to the other derivatives, highlighting the role of substituent-driven modulation of ligand–protein interactions (Mardirossian et al. 2021).

## NMA-based molecular dynamics Results

### MD V. parahaemolyticus between carvacrol/thymol derivatives

**Fig. 5:**
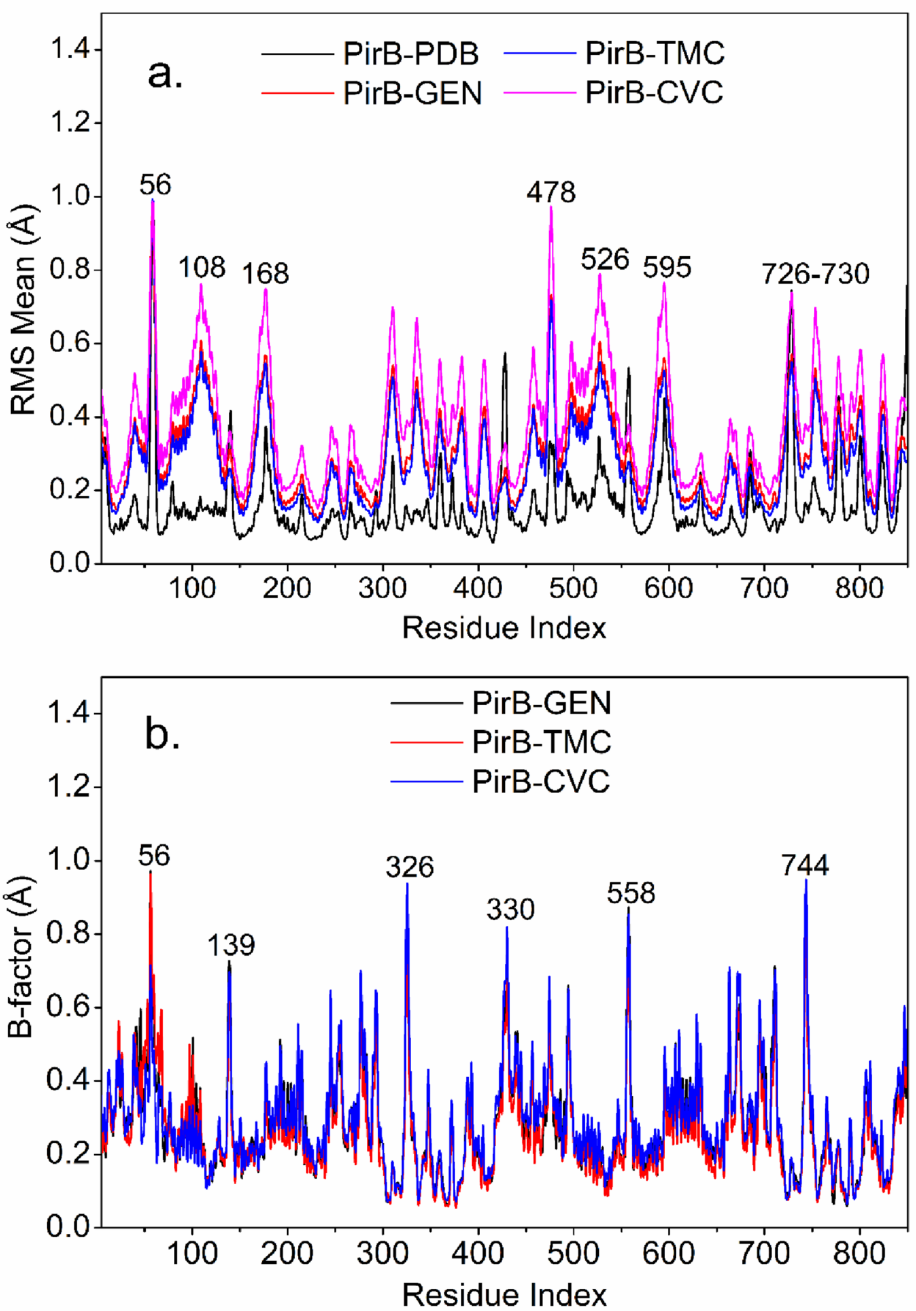
Based MD, **(a)** mode analysis (NMA) showing intrinsically flexible regions of the PirB (vp) subunit (black line), GEN (red line), TMC (blue dark) and CVC (pink line) and **(b)** Comparison of deformability in amino acid residues of the PirB (vp) protein, in complex with GEN (black line), and in the PirB/TMC (red line) and PirB/CVC (blue dark) complexes

Molecular dynamics (MD) simulations based on normal mode analysis (NMA) are used to estimate conformational changes over time in molecular systems (Vidal-Limón et al., 2022). For the antibacterial protein PirB (PDB 3 × 0U, black line) in the presence of the inhibitor GEN (red line) and the complexes with the best molecular docking scores, PirB/TMC (blue dark line) and PirB/CVC (pink line), see Fig. 5a, the mean Cα RMS value (in Å) was evaluated. The results indicated that the PirB protein exhibited an average RMS ranging from 0.0 to 1.0 Å.

Figure 5a illustrates residual analyses showed that the amino acid sequence 54–62 exhibited Cα conformational variations close to 1 Å, while regions corresponding to amino acids 168–180, 474–478, 526 595 and 726–730 exhibited more significant fluctuations, with RMS ranging from 0.1 to 1 Å. The data from the complexes, overlaid on the graph in Fig. 5b, indicate comparable flexibility profiles in relation to the free protein, particularly within regions associated with the binding site. These observations suggest that ligand binding does not markedly alter the overall pattern of collective motions. However, within the framework of Normal Mode Analysis, such results should be interpreted as qualitative indications of intrinsic flexibility rather than evidence of competitive binding or dynamic behavior.

These results indicate that TMC and CVC are promising molecules with potential antibacterial activity against *V. parahaemolyticus*, since the RMSD values remain below 2.0 Å, demonstrating good structural stability of the protein in the presence of the ligands (Firdous et al. 2023).

With regard to deformability, this property was evaluated in the PirB/TMC (red line) and PirB/CVC complexes, in comparison with the GEN inhibitor. Discrete peaks of deformation were observed at residues 56 and 325, in the presence of TMC and CVC, respectively, with RMS values close to 1.0 Å, see Fig. 5b. These results indicate that both compounds induce minimal conformational changes in the PirB protein compared to GEN.

**Fig. 6:**
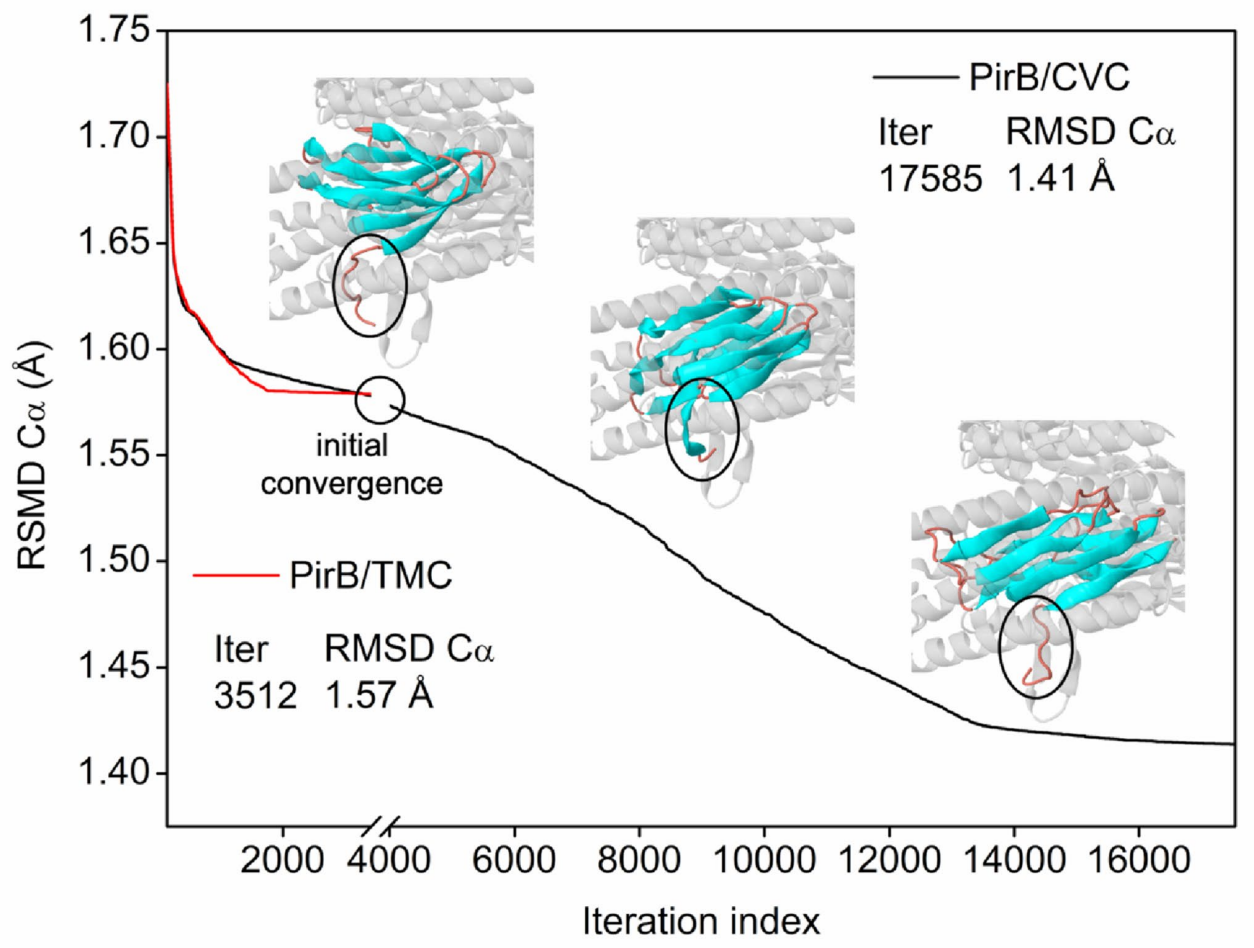
The TMC and CVC (black and red line)/PirB (VP) complex converged to a final RMSD

Figure 6 illustrates the analysis of the root mean square deviation (RMSD) of Cα atoms throughout the simulation iterations allowed for the evaluation of the structural convergence of the PirB/TMC and PirB/CVC complexes relative to the initial protein structure. At the start of the simulation (0 iterations), the complexes exhibited a conformational deviation of 1.72 Å relative to the co-crystallized protein, reflecting the initial structural adjustment of the system. As the simulation progressed, a gradual convergence of the simulated structures toward the reference crystallographic state was observed. After 17.585 cycles, the PirB/CVC complex reached an RMSD of 1.41 Å, indicating conformational stabilization, while the PirB/TMC complex exhibited an RMSD of 1.57 Å, demonstrating comparable structural behavior between the systems.

RMSD values ranging from 1.0 to 2.0 Å are generally indicative of minimal conformational changes, suggesting that structural integrity was preserved during the simulation (Armstrong et al. 2016). In this context, the results obtained indicate that all complexes fall within ranges typically associated with structurally coherent conformations, supporting the conformational consistency of the protein–ligand interactions, Fig. 6. However, within the framework of Normal Mode Analysis, these findings should be interpreted as qualitative indicators of intrinsic flexibility rather than direct evidence of thermodynamic stability.

As shown in Fig. 6, among the systems evaluated, the PirB/TMC complex exhibited the lowest structural variation, reaching convergence in approximately 3.512 iteration cycles, reflecting an efficient structural accommodation process. In contrast, the PirB/CVC complex reached convergence only after more than 17.000 cycles, indicating a slower stabilization of the system. Despite these differences in the number of cycles required, the final RMSD values showed no substantial deviation among the complexes, reflecting a similar structural behavior across the evaluated targets.

Thus, the introduction of aliphatic chains combined with halogen substituents, such as the chlorine atom present in CVC and TMC derivatives, leads to changes in the physicochemical properties of the molecules, particularly an increase in hydrophobicity (Demakov 2023). This modification may favor ligand accommodation within nonpolar regions of the protein cavity, promoting hydrophobic contacts that can contribute to more favorable interaction patterns (Lee et al. 2015).

Although the observed energy differences are modest, these structural adjustments position the TMC derivative within a range compatible with relevant interactions with protein residues, particularly in regions associated with lower flexibility. A similar trend is observed for carvacrol and its derivatives, where structural modification is associated with more consistent interaction profiles.

In this context, although carvacrol exhibits a lower RMSD compared to CVC, its distinct interaction pattern relative to the control and its lower predicted binding affinity suggest comparatively reduced biological relevance, supporting the selection of CVC as the most promising derivative within the group.

Overall, the data indicate that all systems exhibit comparable conformational behavior, with the PirB/TMC complex showing a tendency toward lower structural deviation and faster convergence. Within the limitations of the applied methods, these features suggest a more consistent structural profile, although they should not be interpreted as definitive evidence of enhanced stability.

### MD wTS between carvacrol/thymol derivatives

**Fig. 7:**
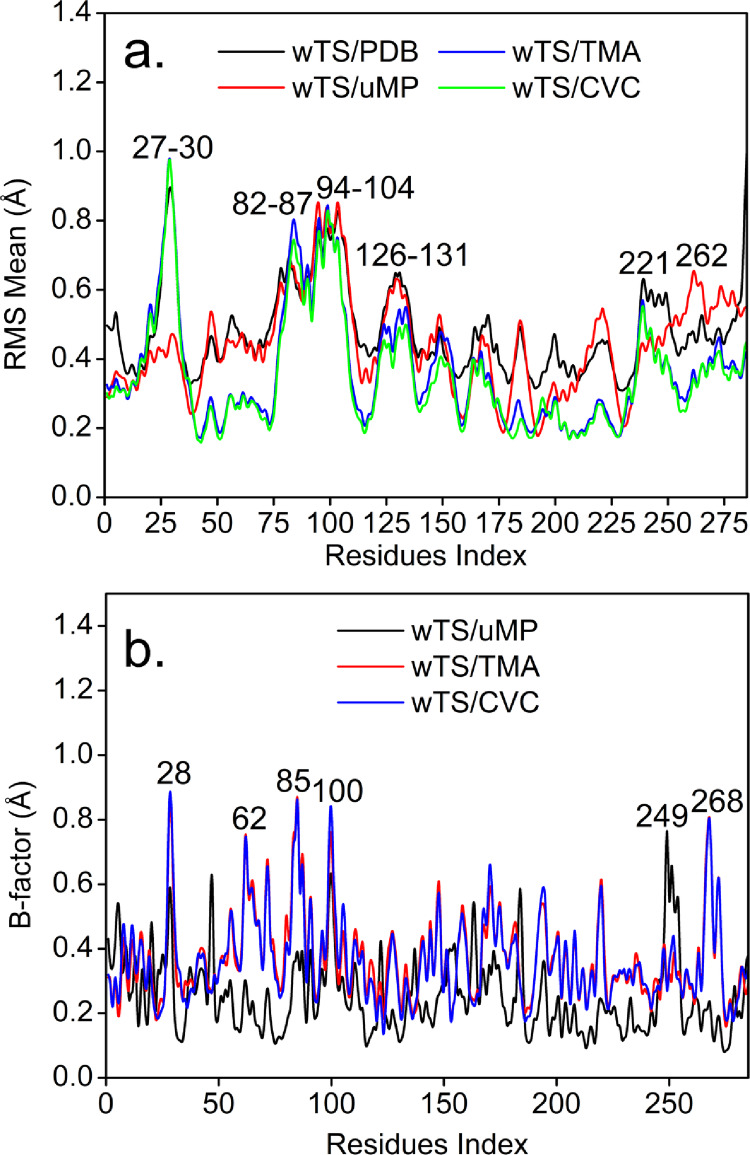
Based MD, **(a)** Mode analysis (NMA) showing intrinsically flexible regions of the WTS subunit (black line), UMP (red line), TMC (blue dark) and CVA (green line) and **(b)** Comparison of deformability in amino acid residues of the WTS protein, in complex with GEN (black line), and in the WTS/TMC (red line) and WTS/CVA (blue dark) complexes

The molecular dynamics of the 7XYJ (wTS) protein were evaluated using the compounds with the lowest interaction energies, given that none of them met the ideal affinity parameters. Throughout the simulation, the Cα atoms exhibited RMS values ranging from 0.0 to 1.0 Å, indicating overall protein stability, see Fig. 7a. Small fluctuation peaks were detected in residues 27–30, while regions 82–87, 94–104, 126–131 and 221–262 exhibited more pronounced conformational variations, with RMS values ranging from 0.5 to 1.0 Å, see Fig. 7b, reflecting localized mobility without compromising the structural integrity of the protein core.

**Fig. 8:**
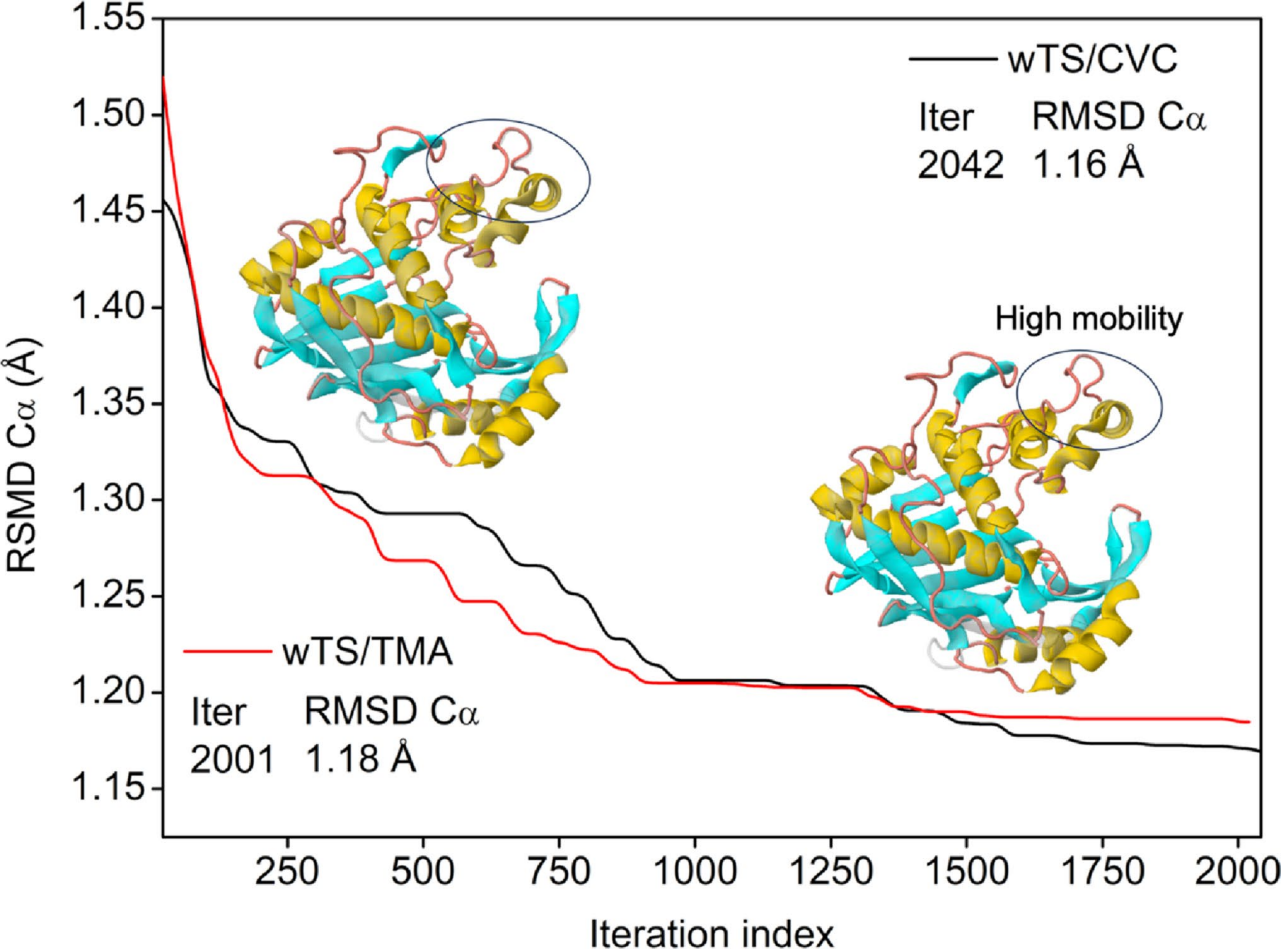
The TMC and CVA (black and red line)/wTS complex converged to a final RMSD

The overlay of the graphs was constructed using data from the complexes, in a manner analogous to the analysis performed for antibacterial activity. The results indicate that the ligands are positioned within the same binding region, exhibiting Cα flexibility profiles comparable to those of the free protein (see Fig. 8). This behavior suggests that ligand binding does not substantially alter the overall pattern of collective motions.

In addition, the observed RMSD values below 2.0 Å indicate limited structural deviation relative to the reference conformation. However, within the scope of the applied methods, these findings should be interpreted as indicators of structural consistency rather than direct evidence of dynamic stability. In this context, the results are consistent with the docking predictions and support the potential of the evaluated derivatives as candidates for further investigation.

In the convergence analysis of the wTS/CVC (red line) and wTS/TMA complexes, compared to the native state of the protein, the derivatives exhibited behavior consistent with that observed in the complexes with PirB, Fig. 8. At the initial stage of the analysis, the complexes exhibited conformational deviations of approximately 1.5 Å relative to the co-crystallized protein, consistent with structural adjustment upon ligand accommodation. As the analysis progressed along the sampled conformational space, the structures showed a tendency toward reduced deviation from the reference crystallographic state. Around 2.041 cycles, the wTS/CVC complex reached an RMSD of 1.16 Å, while the wTS/TMA complex exhibited an RMSD of 1.18 Å after 2.001 cycles (Fig. 8), indicating comparable structural convergence between the systems.

Within the framework of the applied method, these RMSD values reflect limited conformational variation relative to the reference structure and should be interpreted as indicators of structural consistency rather than explicit evidence of time-resolved dynamic stabilization. (Armstrong et al. 2016).

Thymidylate synthase functions as a homodimer, and its intrinsic flexibility including inter-subunit communication and structural rearrangements may influence ligand accessibility and binding affinity. Within the framework of Normal Mode Analysis (NMA), such features are interpreted as collective low-frequency motions rather than explicit time-dependent dynamics. In this context, regions corresponding to active-site loops appear to contribute to conformational adaptability, consistent with their established role in modulating substrate binding and catalytic function through transitions between open- and closed-like states. However, these motions represent approximations of structural flexibility and should not be interpreted as fully resolved dynamic trajectories.

## Ecotoxicological analysis of carvacrol/thymol derivatives

### Hydrophilicity and persistence assessment

 As previously described, the data obtained through the ecotoxicological prediction tools available in the JANUS software were systematically organized in Table S3. This description encompasses both physicochemical properties, including the octanol-water partition coefficient (logKow) and solubility in aqueous medium, and ecotoxicological parameters, such as environmental persistence and lethal and effective concentrations (LC_50_/EC_50_) (Roberto et al. 2025).

In this context, evaluating the hydrophilic character of molecules is a central aspect of ecotoxicological analysis, since the octanol/water partition coefficient (logKow) is widely recognized as one of the main descriptors influencing the environmental and biological behavior of chemical substances (De Sousa et al. 2025a, b). This parameter is directly associated with the distribution of the compound between aqueous and lipid phases, and is crucial for processes such as bioaccumulation, environmental mobility, and toxicity potential in different ecological compartments (De Sousa et al. 2026).

The evaluated compounds presented log Kow values ranging from 3.38 (thymol) to 4.01 (CVA and TMA), indicating an intermediate profile between hydrophobicity and hydrophilicity. This range suggests a relevant tendency for interaction with organic phases, such as biological tissues and sediments, although with a lower bioaccumulation potential than that observed in highly hydrophobic substances (logKow > 5), see Table S3 (Arnot and Gobas 2006; Mackay and Fraser 2000). The similarity of logKow values between structurally related molecules, within a narrow range, suggests that the subsequent properties are strongly modulated by the substituents introduced into the base structure of carvacrol and thymol (Mardirossian et al. 2021).

The aqueous solubility behavior is consistent with this interpretation. It is observed that pairs of molecules containing equivalent substituents exhibit similar solubility values, while differing more markedly in relation to the other analogues: carvacrol (1243.79 mg/L) and thymol (896.91 mg/L); CVA (76.60 mg/L) and TMA (74.39 mg/L); CVB (382.63 mg/L) and TMB (355.75 mg/L); and CVC (3.05 mg/L) and TMC (3.04 mg/L), see Table S3.

Thus, it can be inferred that structural modifications, such as the introduction of halogenated atoms (CVC and TMC), significantly influence electronegativity and intermolecular interactions, resulting in a marked reduction in aqueous solubility. This effect appears to be associated with a decrease in the compound’s solvation capacity in aqueous solution, especially in the absence of compensatory hydrophilic groups (Cronin and Schultz 1997). On the other hand, such modifications have a relatively limited impact on logKow, since they do not substantially alter the overall balance between the hydrophilic and lipophilic phases (Guillory, 2003).

Thus, the previously discussed physicochemical properties directly influence the environmental behavior of compounds, especially regarding their persistence in different environmental compartments (Arnot and Gobas 2003). The combination of moderate hydrophobicity (log Kow between 3.38 and 4.01) and significant variations in aqueous solubility conditions the preferential distribution of these molecules among the aquatic, sedimentary, and soil phases.

In the aquatic compartment, a lower relative tendency for environmental permanence was observed, with a maximum persistence time of up to 19 days, suggesting a greater susceptibility to biodegradation in this medium. Conversely, as aqueous solubility decreases, the tendency to partition into non-aqueous phases increases, resulting in reduced bioavailability in the water column and greater retention in adjacent compartments, especially in the sediment (Cousins and Mackay 2000). In this context, all the derivatives evaluated (CVA, CVB, CVC, TMA, TMB and TMC) showed extremely high persistence in the sedimentary compartment, with times exceeding 180 days, indicating a strong affinity for solid matrices and potential for accumulation in this environment, see Table S3.

The pairs of compounds also showed similarity in persistence behavior, demonstrating a strong inverse correlation between aqueous solubility and residence time in the sediment. In this context, CVA and TMA (201 days), CVB and TMB (561 days), and CVC and TMC (987 days) showed a progressive increase in sediment persistence values as solubility decreased, see Table S3.

This behavior indicates a high affinity of these compounds for the organic matter present in the sediment, which favors sorption and retention processes and, consequently, reduces availability for degradation. This pattern is consistent with environmental models that associate low aqueous solubility with a greater tendency to accumulate in sediments and slower degradation rates in this compartment (Davis et al. 2024; Park and Erstfeld 1999).

In contrast, in the soil compartment, a distinct behavior was observed, with an absence of significant persistence, varying between 5 and 70 days (therefore, < 120 days), see Table S3. Nevertheless, the same general trend of an inverse relationship between solubility and persistence was maintained, although less pronounced when compared to what was observed in the sediment, suggesting a lower retention capacity and greater dynamics of transformation in this environmental compartment (Cousins et al. 2019).

In general, the results indicate that small structural modifications, such as the introduction of hydrophobic or halogenated substituents, can significantly alter the dynamics of evaluating molecular properties, allowing extrapolations between solubility and persistence an approach frequently employed in estimating the environmental behavior of substances not experimentally characterized (Ellis et al. 2002).

Carvacrol and thymol showed low environmental persistence, which is consistent with the phenolic nature of these compounds and their greater susceptibility to degradation (Mukherjee et al. 2024). However, the introduction of short aliphatic chains, in isolation, should not be sufficient to promote drastic increases in environmental persistence profiles. In general, the effect of these chains on physicochemical properties, such as hydrophobicity and persistence, is typically incremental, since each methylene group contributes additively to the gradual increase of these properties (Mansouri et al. 2018).

In this sense, the results suggest that the observed changes may be associated not only with increased hydrophobicity, but also with the absence of polar groups capable of counterbalancing the structural modifications introduced by aliphatic chains, especially in CVC and TMC derivatives, which also exhibit chlorine atom substitution (Bhavani et al. 2024). This combination of factors may explain the more pronounced effects observed in terms of reduced solubility and increased persistence among the compounds evaluated (Ellis et al. 2002).

### Acute and chronic toxicity

The evaluation of LC_50_/EC_50_ values of carvacrol and thymol-derived compounds indicates significant variation in acute toxicity among the different test organisms. For the fish population, concentrations ranged from 0.9797 mg/L (CVC) to 9.18 mg/L (CVB), generally suggesting a moderate toxicity profile. The exception is the CVC derivative, which has a value below 1 mg/L, placing it at the threshold of high acute toxicity, see Table S3.

For D. magna, the results show greater sensitivity compared to fish, with LC_50_ values ranging from 0.3219 mg/L (TMC) to 4.48 mg/L (thymol), see Table S3. In this context, the CVC and TMC derivatives stand out as the most toxic compounds, with values below 1 mg/L, classifying them as highly toxic to this organism. This pattern is consistent with the recognized susceptibility of microcrustaceans to organic compounds, particularly those with a higher affinity for cell membranes and a greater potential for interaction with lipid structures (Connors et al. 2022).

The producer level of the trophic chain was the most sensitive among the bioindicators evaluated. EC_50_ values ranged from 0.04009 mg/L (CVC) to 13.95 mg/L (thymol), showing a wide variation in the toxic potential of the compounds in relation to algae, see Table S3. Again, the CVC derivative stood out for presenting extremely high toxicity, while the compounds of origin maintained higher effective concentration values, indicating a lower relative impact on this trophic level.

Regarding chronic toxicity, the results followed a similar pattern to that observed in acute analyses, mostly falling within the spectrum of moderate toxicity. Exceptions to this behavior were identified for fish, while D. magna and green algae remained predominantly within the range of moderate toxicity. For fish, chronic concentration (ChV) values ranged from 0.0066 mg/L (TMC) to 0.4101 mg/L (thymol), making this organism the most sensitive in the chronic exposure scenario. Furthermore, it was the only group to present values explicitly within the high toxicity threshold, with particular emphasis on CVC (0.0076 mg/L) and TMC (0.0066 mg/L), see Table S3.

As mentioned above, the pattern observed in fish is not repeated in other organisms, indicating a relevant difference in the ecotoxicological response to chronic exposure among the trophic levels evaluated. D. magna showed ChV values ranging from 0.1428 mg/L (CVA) to 2.67 mg/L (TMB), remaining entirely within the range of moderate toxicity. Similarly, the results obtained for green algae showed ChV values between 0.1247 mg/L (CVC) and 1.89 mg/L (thymol), also within the range of moderate toxicity, see Table S3.

For both D. magna and green algae, the compounds CVC and TMC did not show the same pattern of high toxicity observed in fish, suggesting that the toxicological profile of these molecules may be dependent on the organism evaluated. In addition to the already discussed influence of structural modifications, this behavior may be related to differences in the affinity of compounds for organic tissues and membrane structures, especially in more complex organisms, which may favor adverse effects in prolonged exposures (Spurgeon et al. 2020).

Despite strong indications of extrapolation in the prediction of the ecotoxicological properties of carvacrol and thymol-derived compounds, the toxicological behavior observed in both exposure scenarios remains consistent with well-documented patterns in the literature (Aurisano et al. 2019).

The introduction of radicals containing chlorine atoms has historically been a controversial topic in the scientific community due to the ambiguity of their effects, as discussed by (De Sousa et al. 2026; Henschler 1994). In general terms, the toxicity associated with chlorination is often attributed to increased lipophilicity and, consequently, to intensified interactions with biological membranes. However, in the present dataset, a proportional increase in lipophilicity is not observed that could, in isolation, justify the significant increases in persistence and the marked reductions in solubility (Mardirossian et al. 2021).

This result supports the hypothesis that the presence of the chlorine atom contributes mainly to the decrease in the overall polarity of the molecule and its solubility in aqueous medium (Lunghini et al. 2020). However, in the absence of polar functional groups capable of compensating for this structural alteration particularly in CVC and TMC derivatives, where chlorine is associated with aliphatic chains an imbalance of physicochemical properties occurs, which may be related to the extrapolated behavior observed in the predictions.

This pattern is also reflected in ecotoxicological assessment, where compounds originally considered to have low toxicity, such as carvacrol and thymol, whose biological activities are widely described in the literature (Imran et al. 2022), begin to show substantial alterations after structural modifications involving chlorination. Although this type of substitution is frequently associated with an increase in the potential for biological activity in certain pharmacological contexts, the literature also highlights the need for caution, since such modifications can unpredictably alter the overall toxicological profile of the molecules (Chiodi and Ishihara 2023).

## Conclusion

Advanced computational analyses indicate that thymol and carvacrol derivatives interact effectively with target proteins associated with antibacterial and antiviral activity. In terms of antimicrobial activity, CVC and TMC stood out, exhibiting high structural stability and minimal conformational changes during the simulations. For antiviral activity, the derivatives occupied hydrophobic regions overlapping with the native ligand, suggesting potential steric blocking and restriction of the natural substrate’s access to the active site.

Predictive ecotoxicological analysis highlighted CVC and TMC derivatives as the most critical, in which halogenation promoted significant increases in persistence and toxicity. However, such variations may reflect limitations in extrapolating the predictive model, since the isolated contribution of halogenated atoms would hardly justify such a marked alteration in the toxicological profile of these base molecules.

Taken together, the data indicate that modified phenolic monoterpenes, particularly CVC and TMC, show potential as bioactive candidates. Given the predictive nature of computational simulations, complementary in vivo and in vitro studies are needed to confirm the ability of these derivatives to modulate antibacterial and antiviral activity, contributing to therapeutic strategies aimed at controlling WSSV and AHPND in shrimp farming.

In this context, this study establishes a foundation for future research aimed at validating the biological activity of promising compounds such as CVC and TMC, as well as exploring new structural modifications capable of enhancing the identified molecular interactions.

## Supplementary Information

Below is the link to the electronic supplementary material.

Supplementary Material 1

Supplementary Material 2

Supplementary Material 3

Supplementary Material 4

## Data Availability

All survey data are available exclusively in the supplementary material.
