# Supplementary material for: Molecular and structural insights into carvacrol and thymol alkylated derivatives targeting WSSV and AHPND-causing Vibrio parahaemolyticus
Source: Arch Microbiol. 2026 Jun 6;208(8):420. doi: 10.1007/s00203-026-04982-8 (PMC13242452; doi:10.1007/s00203-026-04982-8)
Supplement: Supplementary file 1 — Supplementary Material 1 [file 203_2026_4982_MOESM1_ESM.docx]

**-- Supplementary material --**

**Molecular and Structural Insights into Carvacrol and Thymol Alkylated Derivatives Targeting WSSV and AHPND-Causing *Vibrio parahaemolyticus***

**Table S1.** Affinity energy values, RMSD, and L–P interaction profiles obtained by molecular docking for carvacrol and thymol derivatives in complexes with the PirB protein.

| **Compd** | **Inter. type** | **Residues / Distances (Å)** |
| --- | --- | --- |
| CVA | Hydrophobic | F244B (3.67), F244B (3.71), F244B (3.63), F244B (3.67)  M247B (3.99), L267B (3.55), T288A (3.82) |
|  | H-bond | T201B (3.69) |
| CVB | Hydrophobic | F244B (3.78), F244B (3.61), F244B (3.65), L267B (3.40), T288A (3.91) |
|  | H-bond | D216A (3.20), R236 (2.31) |
|  | π-Stacking | F244 (4.48) |
| TMA | Hydrophobic | T209A (3.83), F244A (3.51), F244A (3.72), M247A (3.66), L267A (3.33), T288B (3.61), D290B (3.68) |
|  | π-Stacking | F244A (4.34) |
| TMB | Hydrophobic | A213B (3.58), F244A (3.84), M247Z (3.79), T288B (3.46) |
|  | H-bond | T201A (2.41), S205A (2.70), T209A (2.65), N217B (2.40), N217 (2.42) |
|  | π-Stacking | F244A (4.14) |

**Table S2.** Data on ligand-receptor (L-R) interactions in the redocking process of the co-crystallized uMP and molecular docking simulations of the carvacrol/thymol derivatives, via wTS receptor.

| **Compd** | **Inter. type** | **Residues / Distances (Å)** |
| --- | --- | --- |
| uMP | H-bond | H171 (2.41), S191 (1.90), Y233 (2.82) |
|  | Salt bridge | R24 (4.23), R150 (4.17), R151 (4.35), R190 (4.39) |
| Carvacrol | Hydrophobic | I83A (3.42, I83A (3.92), F200A (3.74), F200A (3.73), F200A (3.76) |
|  | H-bond | L196 (2.95) |
|  | π-Stacking | F200A (3.74) |
| CVA | Hydrophobic | L196A (3.74), F200A (3.70), F200A (3.74), F200A (3.72) |
|  | π-Stacking | F200A (3.77) |
| CVB | Hydrophobic | I83A (3.84), W84A (3.77), L196A (3.75), F200A (3.72), F200A (3.94), F200A (3.69) |
|  | H-bond | Q189A (2.41), S191A (2.90), D193A (2.89) |
| CVC | Hydrophobic | W84A (3.91), L196A (3.73), F200A (3.73), F200A (3.70), F200A (3.72) |
|  | π-Stacking | F200A (3.78) |
| Thymol | Hydrophobic | I83A (3.52), F200A (3.77), F200A (3.70), F200A (3.61) |
|  | H-bond | L196A (2.10) |
|  | π-Stacking | F200A (3.80) |
| TMA | Hydrophobic | I83A (3.67), I83A (3.69), F200A (3.64), F200A (3.37) |
| TMB | Hydrophobic | W84A (3.56), W84A (3.98), Y110A (3.64), F200A (3.71) |
|  | H-bond | N201A (3.86), N201A (2.90) |
| TMC | Hydrophobic | I83A (3.83), I83A (3.71), I83A (3.49), I84A (3.64), F200A (3.52) |
|  | π-Stacking | F200A (5.04) |

**Table S3.** Values ​​obtained through ecotoxicological prediction screening of carvacrol and thymol compounds and their respective derivatives

|  | | | **Persistence (days)** | | | **Acute (mg/L)** | | | **Chronic (ChV, mg/L)** | | |
| --- | --- | --- | --- | --- | --- | --- | --- | --- | --- | --- | --- |
| **Compd** | **logKow** | **Solub (mg/L)** | **Water** | **Sediment** | **Soil** | **Fish** | **DM** | **GA** | **Fish** | **DM** | **GA** |
| **Carvacrol** | 3.49 | 1243.79 | 2 | 19 | 5 | 4.85 | 4.04 | 5.4200 | 0.2849 | 0.2531 | 0.8301 |
| CVA | 4.01 | 76.6 | 19 | 561 | 20 | 1.22 | 2.33 | 0.78790 | 0.1270 | 0.1428 | 0.1566 |
| CVB | 3.59 | 382.63 | 8 | 201 | 20 | 9.18 | 1.84 | 2.68000 | 0.1586 | 2.64 | 0.2555 |
| CVC | 3.92 | 3.05 | 19 | 987 | 70 | 0.9797 | 0.3702 | 0.04009 | 0.0765 | 0.1591 | 0.1247 |
| **Thymol** | 3.38 | 896.91 | 2 | 19 | 5 | 4.68 | 4.48 | 13.95 | 0.4101 | 1.99 | 1.89 |
| TMA | 4.01 | 74.39 | 19 | 561 | 20 | 1.2 | 2.18 | 0.7388 | 0.1289 | 0.1473 | 0.1981 |
| TMB | 3.59 | 355.75 | 7 | 201 | 20 | 8.11 | 1.32 | 4.85 | 0.1255 | 2.67 | 0.2801 |
| TMC | 3.92 | 3.04 | 19 | 987 | 70 | 1.89 | 0.3219 | 0.407 | 0.0066 | 0.1517 | 0.1434 |

**Notes:** LogKow - octanol-water partition coefficient; Solub - solubility; ChV - chronic concentration; DM - Daphnia magna; GA - green algae.
