# Supplementary figures and images for: Molecular and structural insights into carvacrol and thymol alkylated derivatives targeting WSSV and AHPND-causing Vibrio parahaemolyticus
Source: Arch Microbiol. 2026 Jun 6;208(8):420. doi: 10.1007/s00203-026-04982-8 (PMC13242452; doi:10.1007/s00203-026-04982-8)

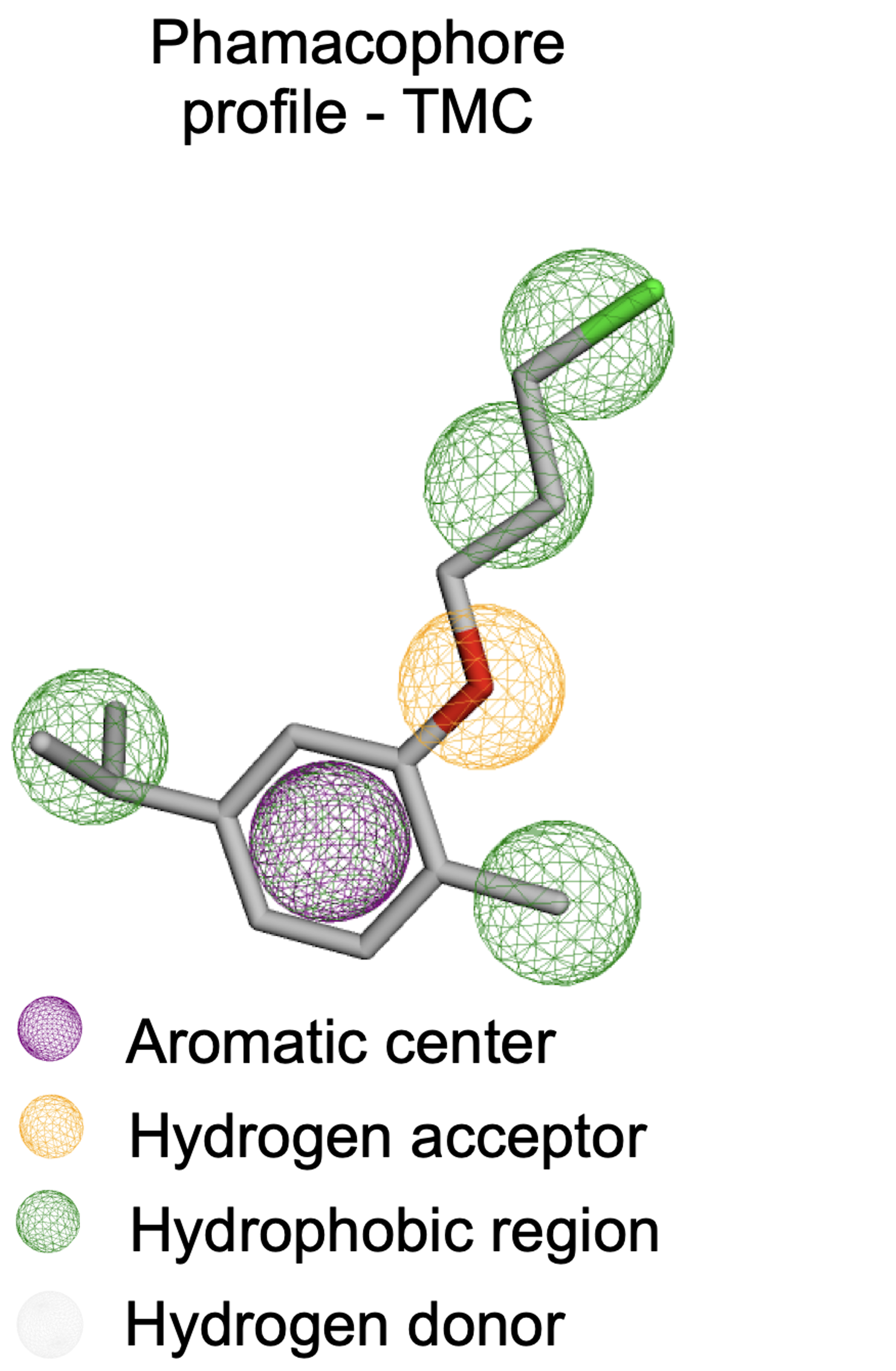

Supplement: Supplementary file 3 — Supplementary Material 3 [file 203_2026_4982_MOESM3_ESM.png]
